# Digital dissection of the head of the rock dove (*Columba livia*) using contrast-enhanced computed tomography

**DOI:** 10.1186/s40851-019-0129-z

**Published:** 2019-06-10

**Authors:** Marc E. H. Jones, David J. Button, Paul M. Barrett, Laura B. Porro

**Affiliations:** 1 Department of Earth Sciences, Natural History Museum, London, SW7 5BD UK; 2 Department of Cell and Developmental Biology, UCL, University College London, Gower Street, London, WC1E 6BT UK

**Keywords:** *Columba*, Jaw muscles, Iodine, Eye muscles, Hyolingual apparatus, Digital dissection

## Abstract

The rock dove (or common pigeon), *Columba livia*, is an important model organism in biological studies, including research focusing on head muscle anatomy, feeding kinematics, and cranial kinesis. However, no integrated computer-based biomechanical model of the pigeon head has yet been attempted. As an initial step towards achieving this goal, we present the first three-dimensional digital dissection of the pigeon head based on a contrast-enhanced computed tomographic dataset achieved using iodine potassium iodide as a staining agent. Our datasets enable us to visualize the skeletal and muscular anatomy, brain and cranial nerves, and major sense organs of the pigeon, including very small and fragile features, as well as maintaining the three-dimensional topology of anatomical structures. This work updates and supplements earlier anatomical work on this widely used laboratory organism. We resolve several key points of disagreement arising from previous descriptions of pigeon anatomy, including the precise arrangement of the external adductor muscles and their relationship to the posterior adductor. Examination of the eye muscles highlights differences between avian taxa and shows that pigeon eye muscles are more similar to those of a tinamou than they are to those of a house sparrow. Furthermore, we present our three-dimensional data as publicly accessible files for further research and education purposes. Digital dissection permits exceptional visualisation and will be a valuable resource for further investigations into the head anatomy of other bird species, as well as efforts to reconstruct soft tissues in fossil archosaurs.

**Electronic supplementary material:**

The online version of this article (10.1186/s40851-019-0129-z) contains supplementary material, which is available to authorized users.

## Introduction

The rock dove (*Columba livia*)*,* also known as the common or domestic pigeon, is one of the most abundant, widely distributed, and morphologically disparate bird species, including over 350 breeds [1, 2], and has a long association with human culture [3]. Careful documentation of variations in rock dove morphology and behaviour were key to the development of evolutionary theory [4] and, more recently, it has become an important model organism in biological sciences, including genetics [5, 6], evolutionary biology [1, 2, 6–8], neuroscience and behaviour [9–13], vision [14], biomimetics [15], developmental biology [16], medicine [17, 18] and biomechanics [19, 20], including feeding studies [21–29].

Several descriptions of the head musculature of *C. livia* have been published [25, 30–33], and the pigeon is frequently chosen as an exemplar of avian anatomy in zoological and veterinary textbooks (e.g., [34, 35]). Descriptions, of varying completeness, are also available for other closely related columbiform species (Table 1), including *Columba palumbus*, the wood pigeon [38], *Didunculus strigirostris*, the tooth-billed pigeon [40], and *Treron phoenicoptera*, the common green pigeon [25, 30]. It has been suggested that columbiform birds are relatively conservative with respect to their pecking and drinking behaviour ([32]: page 2); however, they do exhibit variation in their feeding anatomy and ecological role. Most taxa (e.g., *Columba*, *Streptopelia*) use their jaws primarily to garner loose seeds and grains and to gather twigs for nests; however, they will occasionally feed on insects and fruit [25, 27, 28, 30, 31, 42]. Other species, such as *Duculua aenea*, the Nicobar green imperial pigeon, are entirely fruit-eating and exhibit larger and more complex jaw muscles [25, 30, 40]. A greater understanding of the variation in jaw muscle anatomy is likely to inform our understanding of the variations in skull shape that do exist (e.g., [4, 25, 29, 31, 40, 43, 44]) given their likely relationship to differences in muscle size and arrangement (e.g., [42, 45, 46]). Furthermore, a detailed survey of muscle anatomy will provide key baseline data for biomechanical analyses that could be used to test hypotheses related to ecomorphology, feeding behaviour, and skull mechanics.

**Table 1:** Previous descriptions of jaw, neck, and throat muscles in columbiform birds

Genus	species	Common name	References
*Columba*	*livia*	common pigeon	Burk 1893; Rawal 1970; Bhattacharyya 1980, 1994, 2013; Zweers 1982; Van Gennip 1986 [25, 30–33, 36, 37]
*Columba*	*palumbus*	woodpigeon	Rooth 1953; Barnikol 1953 [38, 39]
*Alopecoenas* (=*Gallicolumba*)	*rubescens*	Marquesan ground dove	Burton 1974 [40]
*Didunculus*	*strigirostris*	tooth-billed pigeon	Burton 1974; Bhattacharyya 1994 [25, 40]
*Ducula*	sp.	imperial pigeon	Bhattacharyya 2013 [31]
*Ducula*	aena	Nicobar green imperial pigeon	Bhattacharyya 1994 [25]
*Ducula*	badia	Hodgson’s imperial pigeon	Bhattacharyya 1994 [25]
*Streptopelia*	“*risoria*”	Barbary dove	Barnikol 1953; Starck and Barnikol 1954 [39, 41]
*Spilopelia* (=*Streptopelia*)	*chinensis*	Indian spotted dove	Bhattacharyya 1994 [25]
*Streptopelia*	*decaocto*	collared dove	Bhattacharyya 1994 [25]
*Treron*	*waalia*	Bruce’s green pigeon	Burton 1974 [40]
*Treron*	*phoenicoptera*	common green pigeon	Bhattacharyya 1994, 2013 [25, 31]
*Zenaida*	*aurita*	zenaida dove	Merz 1963 [42]
*Zenaida*	*asiatica*	white-winged dove	Merz 1963 [42]
*Zenaida* (*=Zenaidura*)	*macroura*	mourning dove	Merz 1963 [42]

Advances in medical imaging and computing power have re-energized interest in vertebrate anatomy and facilitated the creation of three-dimensional (3D) digital anatomical and biomechanical models (e.g. [47–59]). Over the past decade, foundational information from gross dissection has been built on by an increasing number of ‘digital dissections’ using radiographic contrast agents, particularly iodine potassium iodide (I_2_KI) combined with high-resolution micro-computed tomographic (μCT) scanning [60–62], widely known as diffusible iodine-based contrast-enhanced μCT, or diceCT [63]. Unlike gross dissection, diceCT is non-destructive, permits visualization of very small and fragile specimens, and preserves the 3D topology of anatomical structures. Furthermore, digital dissections produced through diceCT can be used to create interactive 3D reconstructions for wider distribution to the public, students, and researchers. Digital dissections have been produced for a range of vertebrates, including fish [64], amphibians [65], non-avian reptiles [66–68], and mammals [69–72], as well as various anatomical regions of some birds [58, 73, 74]. Digital dissections have also been possible with unenhanced μCT [75, 76], magnetic resonance imaging [77], and histological sections [78].

Here, we provide the first 3D digital dissection of the head of *C. livia* and a critical synthesis of previous anatomical descriptions of this model organism. We focus on musculoskeletal anatomy, although our work also covers some aspects of the cranial nerves, glands, and eye. In particular, we use these data in an attempt to resolve discrepancies between earlier anatomical descriptions, providing a crucial first step towards developing a three-dimensional biomechanical model of the pigeon skull. These data will also assist reconstructions of head muscles in non-model birds, as well as contributing to a broader understanding of archosaur jaw muscle evolution ([58, 75, 79–85].

## Materials and methods

A deceased and previously frozen adult specimen of *Columba livia* with a body mass of 242 g was obtained from Jim Usherwood and John Hutchinson (Royal Veterinary College, Hatfield, UK).

*Columba livia* is an anatomically disparate species that includes 350 breeds [1] classified within the clade Columbiformes, which contains approximately 44 genera and 315 species of pigeons and doves [86]. According to analyses of nDNA Columbiformes is the sister taxon to a clade comprising Pteroclidiformes (sandgrouses) + Mesitornighiformes (mesites) [87, 88]. Together, these clades form the Columbimorphae, within the larger group Neoaves [87, 88]. Genome scale analyses place the origin of crown group Columbiformes between 18.9 and 31.3 Ma, within the Oligocene or earliest Miocene [89].

The specimen was CT scanned three times in 2016 at the Cambridge Biotomography Centre (Zoology Department, University of Cambridge): once prior to staining, once after staining, and once after some staining was removed. All scans were performed with a Nikon X-Tek H 225 μCT scanner (Nikon Metrology, Tring, UK) and used a tungsten target, a background medium of air, no filter, and were rendered as 16-bit TIFFs. The first scan, prior to staining, used settings of 80 kV and 230 μA, exposure 1000 ms, 720 projections, and no frame averaging to produce 1946 TIFF slices at 0.113 mm/voxel. The second scan was made after staining. The head was severed between the third and fourth cervical vertebrae and fixed in 4% phosphate-buffered paraformaldehyde solution. It was stained in a solution of 7.5% weight-by-volume I_2_KI for 11 days; the solution was neither refreshed nor agitated during this time. This second scan used settings of 140 kV and 90 μA, exposure 1000 ms, 720 projections, and no frame averaging to produce 1267 TIFF slices at 0.0397 mm/voxel. The third scan took place after some of the stain was removed using three separate changes of ethanol and settings of 120 kV and 250 μA, exposure 500 ms, 3141 projections, no frame averaging, to produce 1603 TIFF slices at of 0.0303 mm/voxel.

**Fig. 1:**
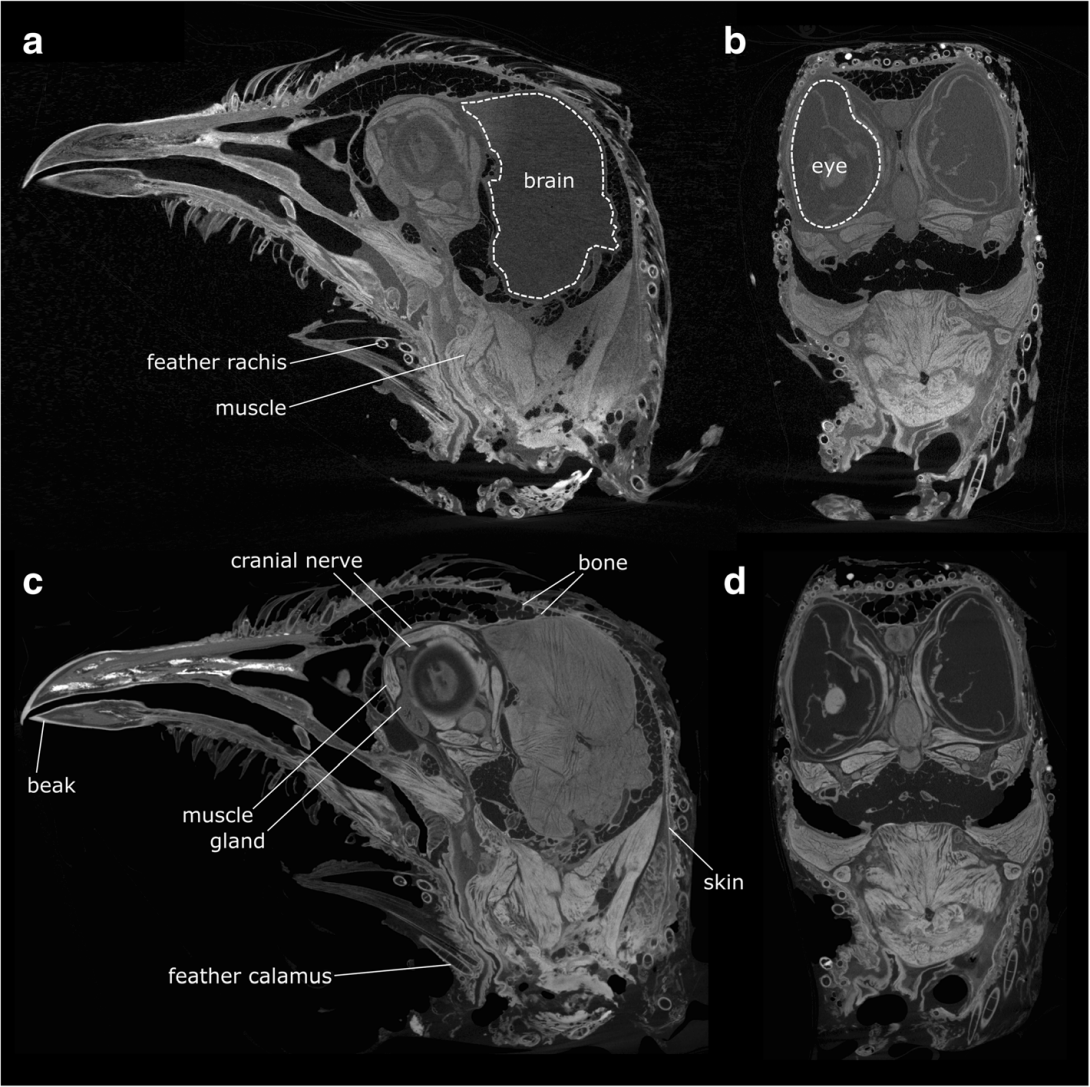
Cross-sections through the Computed Tomography dataset of the iodine stained head of *Columba livia*. **a**,**b**, dataset 2, **c**,**d**, dataset 3. **a** 499 of 879 (parasagittal plane, yz). **b** 1065 of 1678 (coronal plane, xz). **c** 387 of 667 (parasagital plane, yz). **d** 1026 of 1515 (coronal plane, xz)

The μCT scans generated three datasets, each with its own representation of the same specimen. The third dataset provided better representation of some structures (e.g., cranial nerves, ligaments, deep neck muscles) than the second, demonstrating that, in addition to differential absorption of iodine, there must also be differential retention of iodine. The first dataset was used to make a model of the bones, the second was used for the majority of the muscles (Fig. 1a, b), whereas the third was used for the cranial nerves, ligaments, as well as some of the thinnest or deepest muscles (Fig. 1c, d). Muscle fibres and skin readily absorb iodine and therefore appear bright, whereas nerves tend to be darker, but their courses can be identified by careful scrolling between slices. Blood vessels are not always apparent, but when visible they appear as dark hollow structures. Aponeuroses and cartilage do not absorb iodine and, therefore, are not visible in the datasets; however, their shape or location can, to some extent, be inferred from the dark spaces between those tissues that do absorb iodine. Muscle fibre orientation is often visible. The general arrangement of muscles was the same on both sides of the head, but there was some asymmetry in the size and shape of some muscle portions. This variation is probably due to both natural asymmetry and differences in how these pliable structures were resting and compressed during tissue fixation and staining. We did not digitally segment the brain, because digital descriptions and dissections are already available for pigeon neuroanatomy [90, 91].

**Fig. 2:**
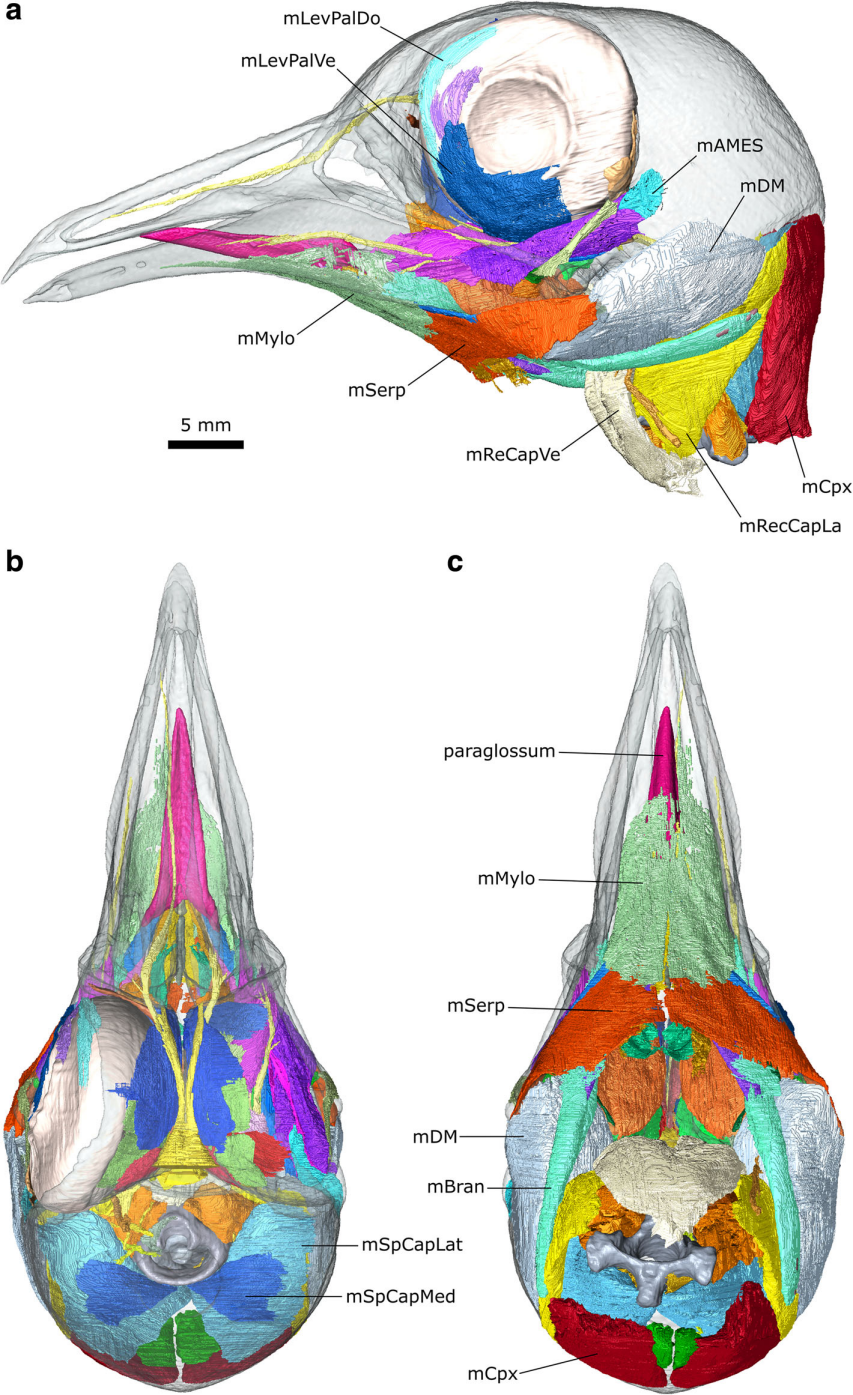
Digital dissection of the head of *Columba livia*. **a** lateral view. **b** dorsal view. **c** ventral view

The μCT datasets were segmented using Avizo 9.2 (Thermo Fisher Scientific, Waltham, Massachusetts, USA) using a mixture of the lasso, draw, interpolation, threshold, and blow tools (Fig. 2). Thresholding with some manual segmentation was used to generate computer models of the bones from the first dataset. Manual segmentation was the primary method used to generate models of the muscles and nerves from the second and third datasets. The blow tool was occasionally used to find the edge of certain structures such as muscle blocks but thresholding was not typically found to be useful. Often the lasso and/or draw tool was used to outline structures every 2–4 slices, and the interpolation tool was applied to capture the volume. All structures were examined, checked, and edited in all three planes. Usually both left and right sides were segmented to aid in identification and interpretation. Anatomical structures were delineated using overall shape, structural variations (e.g., differences in fibre orientations between different muscle masses), and differences in density. The dynamic histogram slider in Avizo was adjusted to enhance visual contrast between different types of soft tissues in each slice to facilitate accurate segmentation. For imaging of the digital models (e.g., Fig. 2), the bone segmentation surface files were smoothed but the muscle segmentations were not (so they faithfully reflect the detail of their segmentation and the original scans).

Three-dimensional surfaces were smoothed (Additional file 4) and exported as wavefront (OBJ) files to create interactive 3D PDFs using Tetra4D Reviewer and Converter (Tech Soft 3D, Oregon, USA) and Adobe Acrobat Pro X (Adobe Systems, California, USA) following previous studies [92, 93]. The smoothing was necessary due to file size constraints. These digital models are provided as supporting information (Additional file 1), and are the basis for the following description.

Here, the muscles are described according to their innervation, homology, origin, path, insertion, and possible function. Following Zweers [32], in the case of one bony attachment, this area is considered to be the origin, whereas in the case of two bony attachments the origin is considered to be the less mobile of the two components (cranium < lower jaw < hyoid). Abbreviations used throughout this study are listed in Table 2.

**Table 2:** Abbreviations used throughout the figures and manuscript text. Bones are in all uppercase. Muscles and glands are in title case following a lower case m or g, ligaments are in title case following a capital L, and specific features are all lowercase with abbreviations separated by a full stop

Abbreviation	Structure
Aponeurosis	Aponeurosis of the mAMEM
AT	atlas
AX	axis
BAS	Basihyale
CERA	ceratobranchiale
CN I	olfactory nerve
CN II	optic nerve
CN III	oculomotor nerve
CN IV	trochlear nerve
CN V	trigeminal nerve
CN V_1_	ophthalmic division of the trigeminal nerve
CN V_2_	maxillary division of the trigeminal nerve
CN V_3_	mandibular division of the trigeminal nerve
CN VI	abducens nerve
CN X	vagus nerve
CN XI	accessory nerve
CN XII	hypoglossal nerve
crt.nuchalis	crista nuchalis
crt.polat.pal	crista posterolateral palatinus
crt.temp	crista temporalis
EPIBR	epibranchiale
for.car	foramen carotid
for.CN X	foramen for the vagus nerve
for.CNXII	foramen for the hypoglossal nerve
for.OccVei	foramen for the occipital vein
fos.temp	fossa temporalis
gHar	gland Harderian
gLac	gland Lacrimal
impr.temp	impressio temporalis
larynx	larynx
L2	ligament 2, ligamentum Occipitomandibule
L5	ligament 5, ligamentum Postorbital
L6	ligament 6, ligamentum Quadratomandibular
LDenBran	ligamentum Dentary-Branchiomandibularis
LOccAt	ligamentum Occipital-Atlantes
mAMEM	musculus Adductor Mandibulae Externus pars Medialis
mAMEMa	musculus Adductor Mandibulae Externus pars Medialis portion a
mAMEMb	musculus Adductor Mandibulae Externus pars Medialis portion b
mAMEP	musculus Adductor Mandibulae Externus pars Profundis
mAMES	musculus Adductor Mandibulae Externus pars Superficialis
mAMP	musculus Adductor Mandibulae Posterior
mBivCer	musculus Biventer Cervicis
mBran	musculus Branchiomandibularis
mCergl	musculus Ceratoglossus
mCerhy	musculus Ceratohyoidues
mCpx	musculus Complexus
mCrico	musculus Cricohyoideus
mDM	musculus Depressor Mandubulae
mFlexCol	musculus Flexor Colli
mGlenglAn	musculus Genioglossus Anterior
mHypOb	musculus Hypoglossus Obliquus
mHypRe	musculus Hypoglossus Rectus
mIntspin	musculus Interspinales
mLevPalDo	musculus Levator Palpebrae Dorsalis
mLevPalVe	musculus Levator Palpebrae Ventralis
mMylo	musculus Mylohyoid
mObDo	musculus Obliquus Dorsalis
MObVe	musculus Obliquus Ventralis
mPPQ	musculus Protractor Pterygoidei et Quadrati
mPstP	musculus Pseudotemporalis Profundus
mPstS	musculus Pseudotemporalis Superficialis
mPtDoAn	musculus Pterygoideus Dorsalis Anterior
mPtDoMe	musculus Pterygoideus Dorsalis Medialis
mPtDoPo	musculus Pterygoideus Dorsalis Posterior
mPtVe	musculus Pterygoideus Ventralis
mPyrLig	musculus Pyramidalis Membrane Nictitans Ligament
mPyrMemNic	musculus Pyramidalis Membrane Nictitans
mQuMemNic	musculus Quadratus Membrane Nictitans
mReCapVe	musculus Rectus Capitis Ventralis
mRecCapDo	musculus Rectus Capitis Dorsalis
mRecCapLa	musculus Rectus Capitis Lateralis
mRecDo	musculus Rectus Dorsalis
mRecLa	musculus Rectus Lateralis
mRecMe	musculus Rectus Medialis
mRecVe	musculus Rectus Ventralia
mSerp	musculus Serpihyoideus
mSpCaMe	musculus Splenius Capitis Medialis
mSpCapLa	musculus Splenius Capitis Lateralis
mStern	musculus Sternohyoides
mStyl	musculus Stylohyoideus
Nic	membrane nictitans
paraglossum	paraglossum
pr.porb	processus postorbitale
pr.zyg	processus zygomatica
QUAD	quadrate
sep.intorb	septum interorbitale
URO	urohyale

## Results

### Basic osteology

Previous descriptions of columbiform skulls include those of Rooth [38] Ghetie [94] Bhattacharyya [30] van Gennip [33] Burton [40], and two of these studies [30, 33] include descriptions of *Columba livia*.

**Fig. 3:**
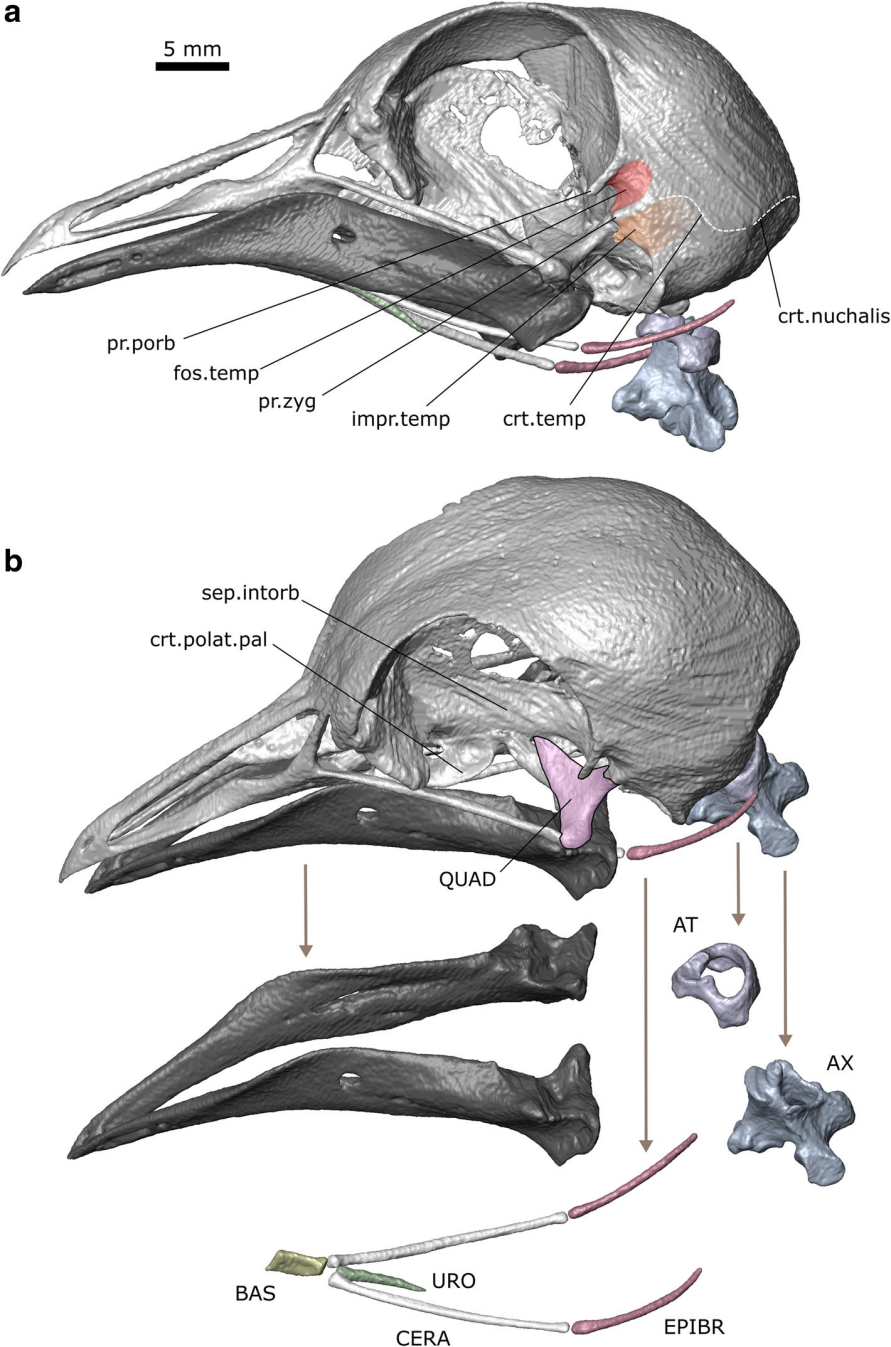
Skull osteology of *Columba livia*. **a** lateral view. **b** dorsolateral view

The skull measures 46.9 mm from the tip of the premaxilla to the basioccipital and 39.7 mm from the bill tip to the ventral lateral corner of the left quadrate (Fig. 3). The skull of *C. livia* is dominated by the braincase and orbits [30, 33]. Connections between the individual bones are variable in the extent and direction of mobility that they permit [29]. Anteriorly the long premaxilla is braced by a prominent midline nasal and the connection between the two bones resembles a hinge [29]. The palatine has two crests extending along its length [33]. Alongside each orbit is a rod-like jugal. The squamosal, parietal, and occipital bones contribute to the braincase vault. The squamosal bears two depressions on its lateral surface: the fossa temporalis anterodorsally and the broader impressio temporalis posteroventrally (Fig. 3a; [33]). Anterior to the fossa temporalis is the prominent postorbital process. Between the two depressions is the zygomatic process. The quadrate is connected to the pterygoid, squamosal, and jugal, and provides the sites of origin for several jaw muscles. As well as forming the craniomandibular joint [30, 33] it is mobile relative to the rest of the cranium and an important component of the kinetic mechanism which allows the upper jaw to be protracted [29, 95].

Each lower jaw comprises six bones (dentary, splenial, surangular, prearticular, articular, and angular) but all except the dentary are fused in adults, such that individual sutures are not visible [33]. Both rami are also fused at the symphysis. In lateral view the anterior half of the lower jaw is at a 30° angle to the long-axis of the posterior half. The posterior surface is flat and triangular (Fig. 3b).

In birds the hyoid skeleton (hyobranchialis apparatus, Additional file 2) comprises three midline elements and a pair of long thin structures (the cornu branchiale) that arise from the junction between the two posterior midline elements (Fig. 3b; [32, 36, 96]). The anterior-most midline element is the cartilagenous paraglossum (=entoglossum). In contrast to some species, the paraglossum in *C. livia* is unpaired (but see [36]). It has an arrowhead-like shape in dorsal view due to the posterolaterally projecting cornua (Fig. 2b; [30]). Its anterior surface is relatively smooth whereas the posterior end bears small spines. The cornified outer ventral layer and tough (but elastic) dorsal layer (described in Bhattacharyya [30]) are both visible in our CT data. The central midline element is the basihyale (=basibranchiale rostrale) and the posterior midline element is the urohyale (=basibranchiale caudale). Both the basihyale and urohyale are flat. Each cornu branchiale comprises a proximal ceratobranchiale, a more distal epibranchiale (=ceratobranchiale II), and in some species a further distal pharyngobranchiale (Fig. 3b; [96]). The latter is present but unossified in *C. livia* and discernible in the CT slices due to the surrounding tissues. Birds exhibit great morphological disparity in the relative proportions of the hyoid skeleton with differences reflecting phylogeny and mode of feeding [36]. The hyoid skeleton of *C. livia* has a rather short basihyale and very long gracile cornua [32, 36]. The paraglossum articulates with the convex anterior surface of the basihyale [32].

### Cranial nerves

**Fig. 4:**
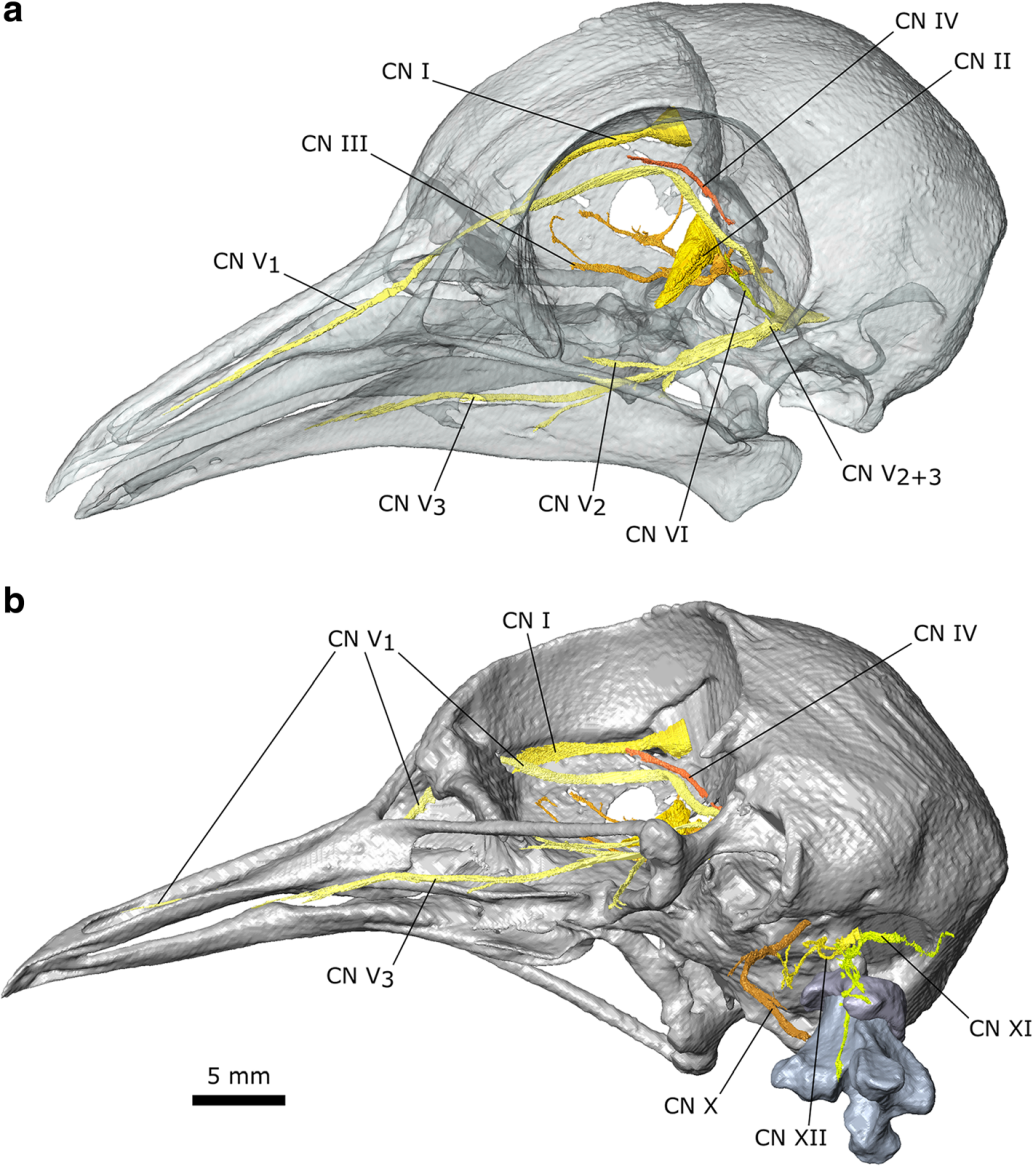
Cranial nerves of *Columba livia*. **a** dorsolateral view of the cranium and lower jaw with bone partly transparent. **b** ventrolateral view of the cranium, atlas, and axis

Several cranial nerves are discernible in the stained pigeon specimen (Fig. 4), which is useful given their roles in evaluating the homology of the adductor musculature [47, 48, 58, 79, 97, 98]. Notable previous descriptions for the cranial nerves of birds include those of Slonaker [99] for the sparrow, Webb [100] for the ostrich, and Ghetie for the turkey [94]. The olfactory nerve (CN I) extends from the olfactory lobe at the anterior of the brain to pass along the underside of the skull roof near the midline (Fig. 2b). The large optic nerve (CN II) exits the brain through the optical canal and into the eye. Two branches of the oculomotor nerve (CN III) are visible within the orbital cavity (passing anteroposteriorly) but whether these two branches are connected, in front of (as in the sparrow: [99]), or behind, CN II is unclear. The trochlear nerve (CN IV) exits the brain lateral to CN II before passing dorsally and curving around the dorsomedial surface of the orbital cavity (Fig. 4a) before innervating the m. Obliquus Dorsalis. It appears to be slightly longer on the right side. The ophthalmic branch of the trigeminal nerve (CN V_1_) exits the braincase ventrolateral to CN II and extends dorsally and then turns sharply to continue anteriorly along the rostrum (Fig. 4a). The maxillary and mandibular divisions of the trigeminal nerve (CN V_2 + 3_) exit the braincase dorsal to the quadrate and divide almost immediately (Additional file 1). The maxillary branch (CN V_2_) appears to be less extensive and is clearer on the left side. It passes down over the quadrate and anteriorly between the internal (m. Pseudotemporalis Superficialis) and external jaw adductor muscles (m. Adductor Mandibulae Externus Medialis). The mandibular branch (CN V_3_) initially lies medial to CN V_2_. It passes ventrally beneath the m. Pseudotemporalis Superficialis and divides into two main divisions. The largest division eventually runs along the medial surface of the lower jaw (Fig. 4a) whereas the smaller medial division passes ventrally between the posterior and medial parts of m. Pterygoideus Dorsalis (Additional file 1). The abducens nerve (CN VI) exits the braincase through the abducens canal close to the m. Rectus Lateralis, which it innervates (Fig. 4a). The vagus nerve (CN X) exits the cranium lateral to the foramen magnum and passes anteroventrally before passing caudally (Fig. 1b). The accessory nerve (CN XI) enters the cranium near the ventral edge of the foramen magnum after passing alongside the atlas and axis (Fig. 1b) medial to the hypaxial muscles. A branch from the CN XI also passes dorsally into the m. Splenial Capitis (initially between its lateral and medial parts). The hypoglossal nerve (CN XII) exits the cranium from two foramina [94, 101] in the basisphenoid located parallel to the midline between the foramen magnum and exit of the CN X (Fig. 4b). The branch from the most anterior foramen passes ventrally between the two most medial heads of the m. Rectus Capitis Dorsalis before turning laterally towards the m. Rectus Capitis Lateralis, whereas the branch from the larger posterior foramen [101] passes between the m. Rectus Capitis Dorsalis and m. Splenius Capitis Lateralis.

### Glands

**Fig. 5:**
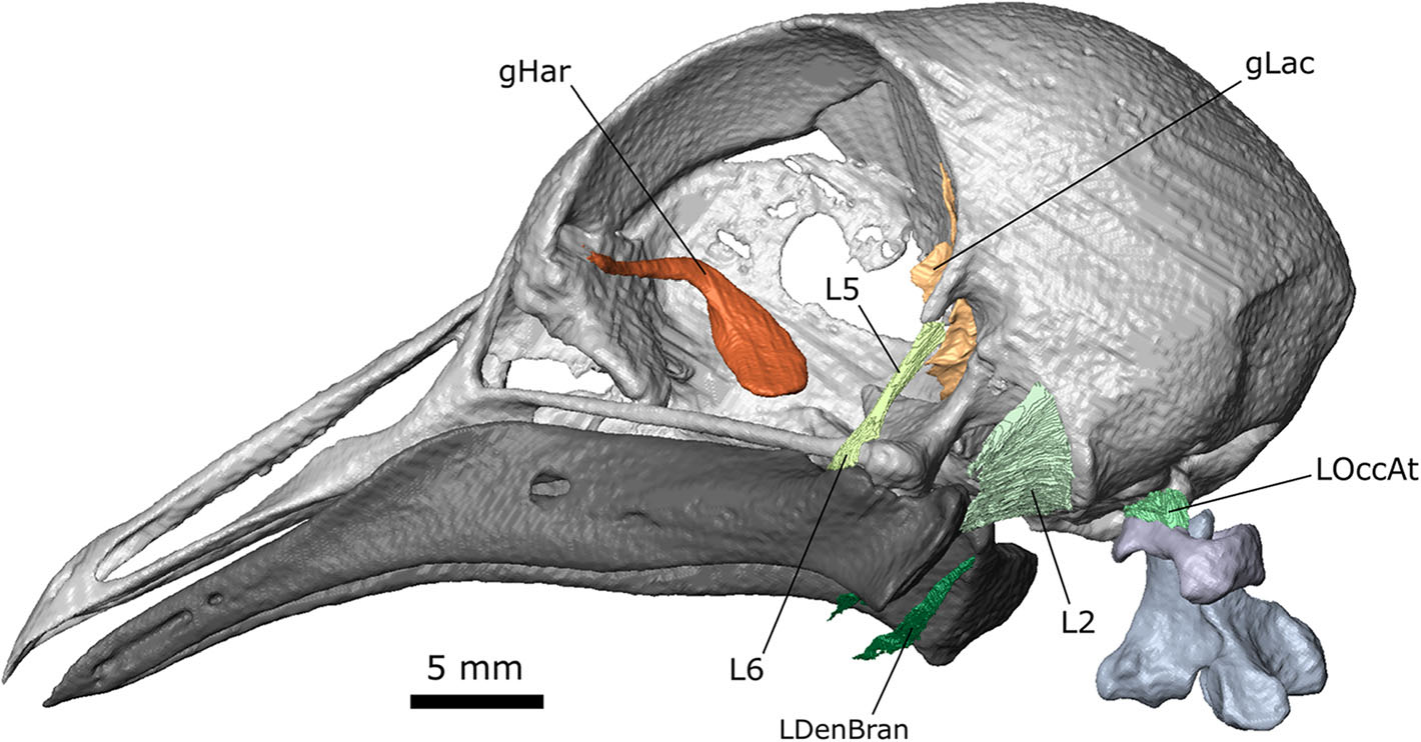
Glands and ligaments of the head of *Columba livia*

Two glands are visible: Harder’s gland and the lacrimal gland (Fig. 5). The main body of Harder’s gland is located against the interorbital bone deep to the m. Rectus Medialis, m. Obliquus Ventralis and m. Pyramidalis Membrane Nictitans [99, 100, 102]. Its duct extends around and over the proximal end of the m. Obliquus Ventralis to the anterior-most boundary of the orbit. The lacrimal gland is much smaller and sits posterior to the eye [99, 102]. Its main body is triangular in cross-section but a duct extends dorsally from it around the body of the eye (Fig. 5).

### Ligaments

Eight extracapsular ligaments have been described in the head of the common pigeon (Fig. 5; [33]; See Table 3). Three of these are visible in the datasets. The broad sheet-like ligamentum occipitomandibule (L2) connects the base of the occiput to the posterior edge of the lower jaw. It has previous been described as laterally thickening [33] and this is evident in the scan. The ligament restricts lateral, ventral, rostral, and medial movements of the lower jaw relative to the quadrate [33]. The long ribbon-like ligamentum postorbital (L5) connects the postorbital process with the jugal bar near the lower jaw. It coordinates movement between the upper and lower jaws [95].

**Table 3:** The skull ligaments of columbiform birds [34] (van Gennip 1986)

Abbreviation	Ligament	Origin	Insertion	Likely role
L1	palatomaxillary	tip of the palatine	maxilla	restricts dorsal movement of the maxilla
L2	occipitomandibule	occiput	posterodorsal edge of the lower jaw	restricts movements of the lower jaw
L3	quadratotemporal	quadrate	suprameaticus	restricts medial movements of the lower jaw
L4	quadratosphenoidal	posteromedial side of the quadrate	parasphenoid	restricts lateral and dorsal directed movements of the quadrate relative to the cranium
L5	postorbital	postorbital	lower jaw	facilitates kinesis of the upper jaw
L6	quadratomandibular lateral	quadrate	lateral side of the lower jaw	restricts rostral and medial movements of the quadrate relative to the lower jaw
L7	quadratomandibular rostral	quadrate	dorsal surface of the lower jaw	restricts lateral movements of the quadrate relative to the lower jaw
L8	jugoprefrontal	jugal	prefrontal	restricts movement of the jugal arch away from the prefrontal

It may also restrict anteroventral movement of the lower jaw (locking it) until contraction of the m. Protractor Pterygoidei et Quadrati rotates the quadrate forwards [26, 27, 29, 33, 95, 103]. The ligamentum quadratomandibular (L6) is short and closely associated with the L5. It spans between the quadrate and lower jaw and is situated just anterior to the jaw joint. This ligament restricts anterior and medial movements of the lower jaw relative to the quadrate [33]. Ligamentous tissue is also present between the ventral and lateral edges of the occipital condyle and the corresponding edge of the atlas (LOccAt). This tissue presumably supports the connection between the skull and neck, and in particular restricts dorsal and mediolateral movements of the head relative to the atlas. A further possible ligament (LDenBran) is visible originating from the posteromedial corner of the lower jaw between the insertion of the m. Pterygoideus Ventralis and m. Depressor Mandibulae. It passes ventrally and folds under the hyoid apparatus, which it presumably supports. This structure does not seem to have been previously described in the literature and it is located posterior to the detailed histological slices presented by Zweers [32].

### Jaw muscles

There have been several previous descriptions of jaw muscles in *Columba livia* [25, 30–33, 37] as well as other columbiform birds (Table 1) [25, 31, 38, 40, 42].

#### m. Adductor Mandibulae Externus (m. AME)

**Fig. 6:**
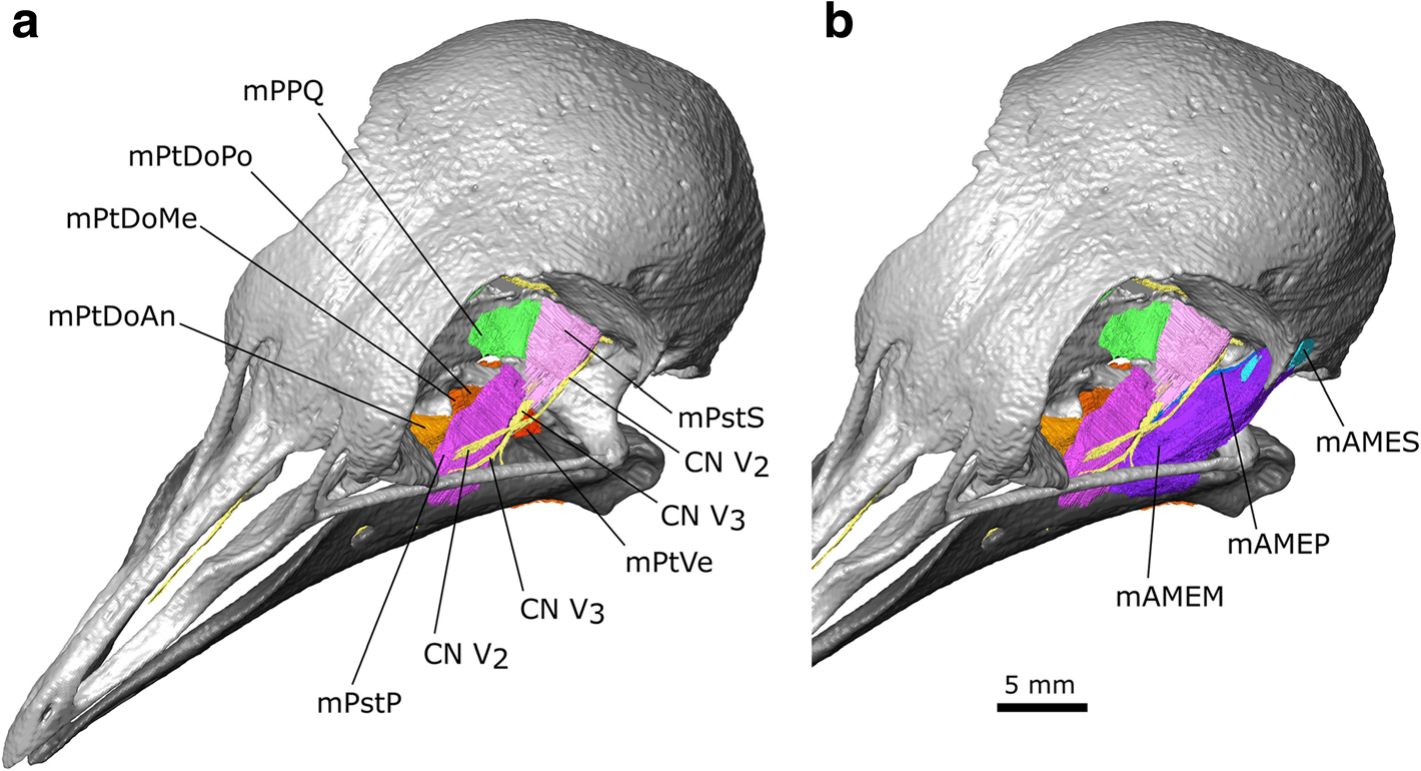
The digital dissection of the left adductor chamber of *Columba livia* showing the location of the adductor muscles relative to the trigeminal nerve (CN V). **a** the external adductor muscles absent **b** the external adductor muscles present

The m. Adductor Mandibulae Externus is innervated by CN V, and in amniotes is usually located between the maxillary (CN V_2_) and mandibular divisions of the trigeminal nerve (CN V_3_) [79, 97]. The m. AME is the most superficial jaw muscle and one of the principal jaw adductors. It has a pennate structure that it generally divided into three parts (pars superficialis, medialis, and profundus), although in many birds the pars superficialis and medialis are difficult to separate [58, 79]. Moreover, there are differences in how these parts are divided and named (e.g., [75, 79, 100, 104]) and it has even been questioned if the homology between them can be established at all [105]. In *Columba livia* the m. AME is located lateral to both the CN V_2_ and V_3_ and there appear to be three distinct parts (Fig. 6; Additional file 1).

#### m. Adductor Mandibulae Externus pars Superficialis (m. AMES)

**Fig. 7:**
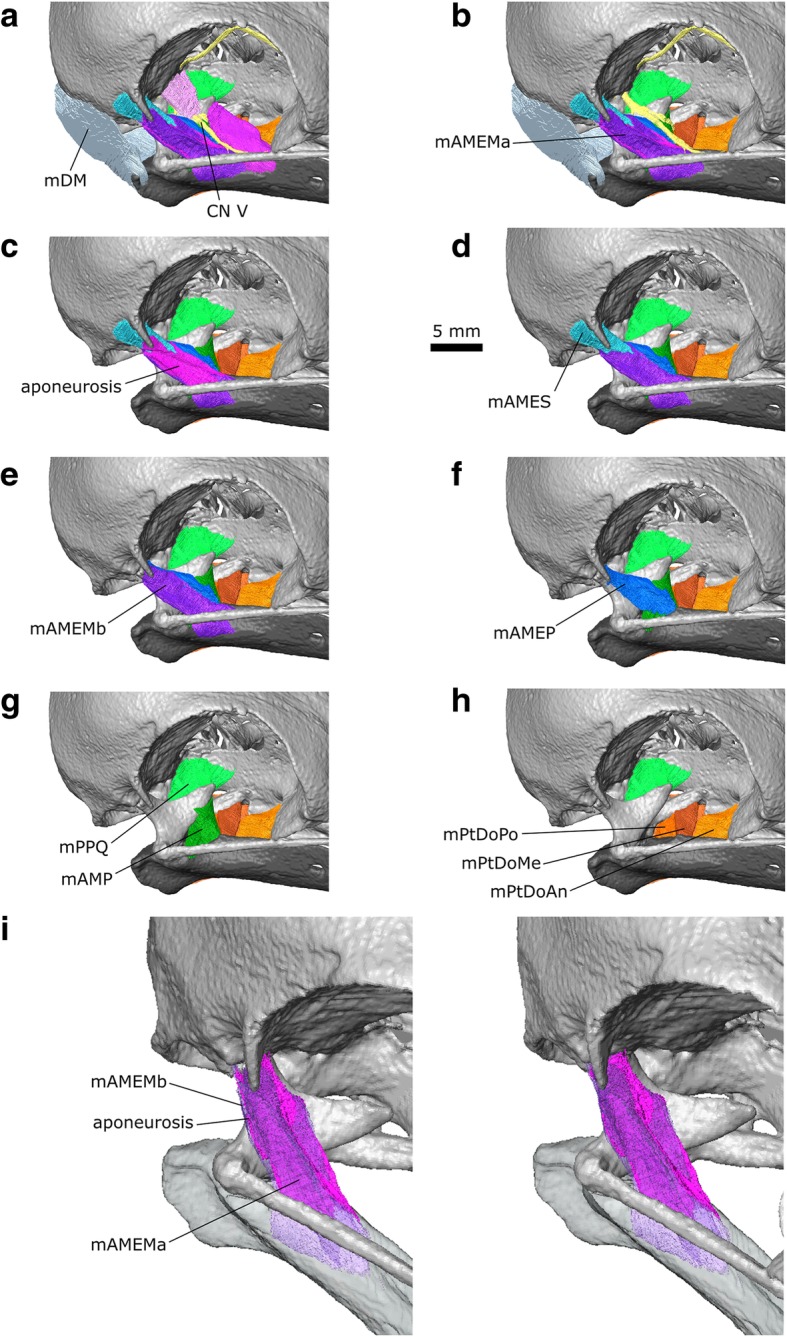
A digital dissection of the right adductor chamber of *Columba livia* in anterodorsal view. **a** to **h** represent increasingly deep dissections. **i** the aponeurosis of mAMEM with the mAMEM partly transparent

**Fig. 8:**
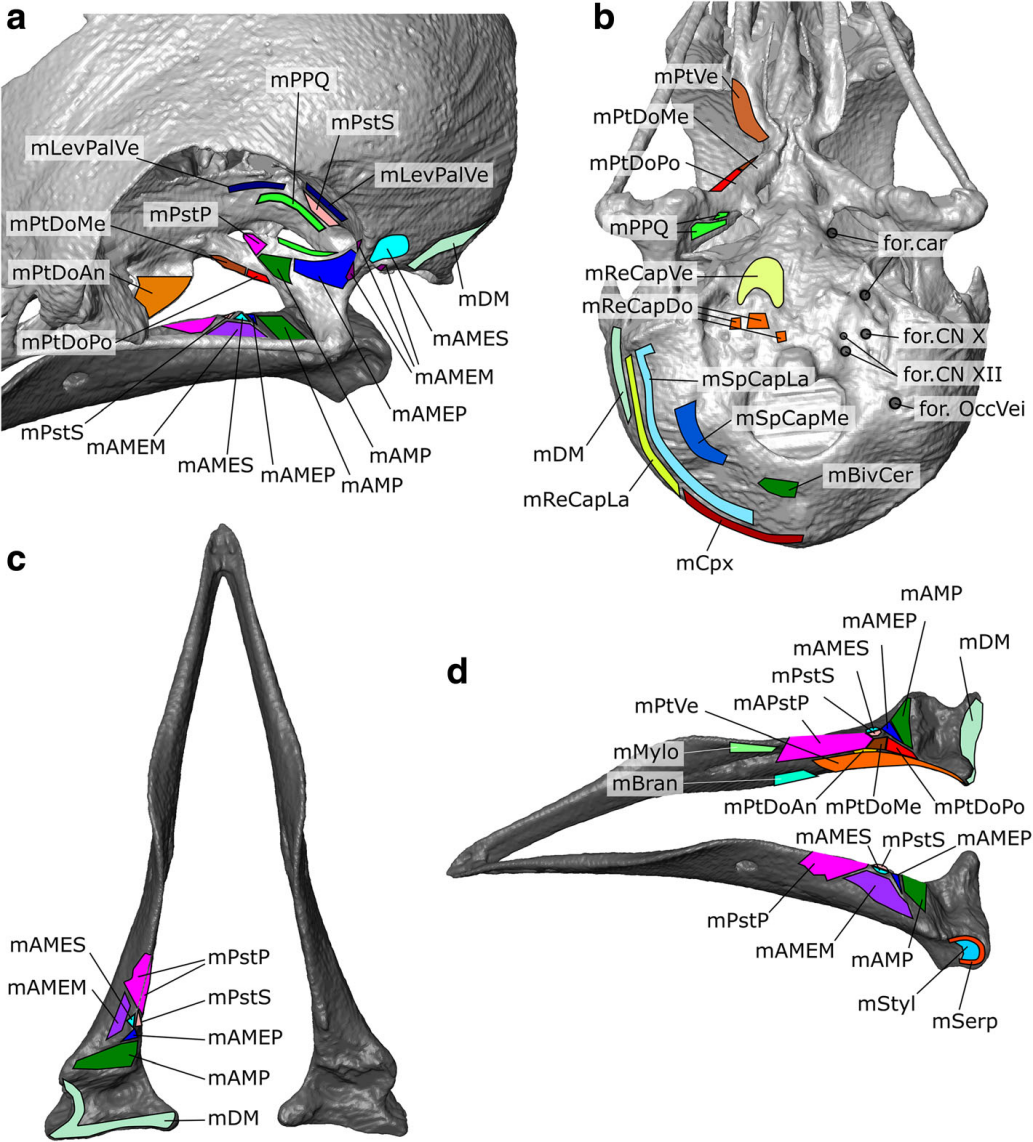
The origins and insertions of the cranial muscles of *Columba livia*. **a** anterodorsal view of the left adductor chamber and orbit **b** ventral view of the cranium **c** dorsal view of the lower jaw **d** dorsolateral view of the lower jaw

**Fig. 9:**
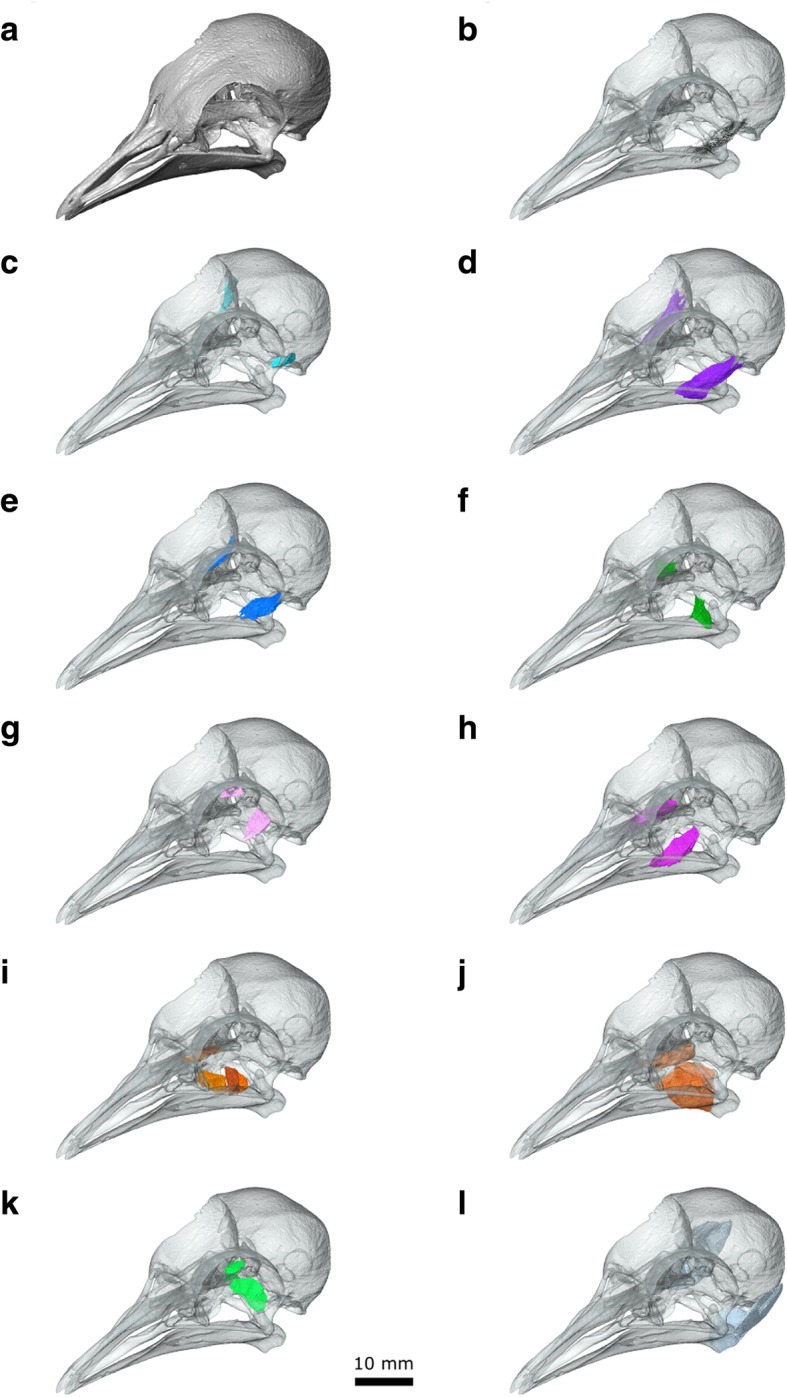
The jaw muscles of *Columba livia* in isolation. **a** the skull shown in opaque. **b** fibres of the mAEME **c** mAMES **d** mAMEM **e** mAMEP **f** mAMP **g** mPstS **h** mPstP **i** mPtDo **j** mPtVe **k** mPPQ **l** mDM

The m. Adductor Mandibulae Externus pars Superficialis lies within the fossa temporalis, which is situated between the prominent postorbital process and the zygomatic process (Figs. 3, 6b, 7d, 8 and 9c). This matches previous descriptions of *Columba livia* [30, 33] but contrasts with descriptions for other members of Columbiformes (e.g., *Columba palumbus* [38]; and various species of *Zenaida* [42]) where some of the muscle within the fossa temporalis was attributed to the m. Adductor Mandibulae Externus pars Medialis. Whether this difference represents genuine species differences or simply differences in dissection approach and interpretation requires a detailed re-examination of those taxa.

**Origin:** From the lateral surface of the squamosal within the fossa temporalis (Fig. 8a; [30, 33]).

**Path:** It passes anteroventrally for only a few millimetres before converging on a point that likely corresponds to the end of an aponeurosis (Figs. 7d, 8c). Ventrally it is bounded by the m. Adductor Mandibulae Externus pars Medialis.

**Insertion:** A small region of the coronoid process of the lower jaw via an aponeurosis (Fig. 8c, d; [30, 33]).

**Function:** Contraction of this muscle rotates the lower jaw upwards. Its’ in vivo activity has not been examined directly ([27, 28]).

#### m. Adductor Mandibulae Externus pars Medialis (m. AMEM)

The m. Adductor Mandibulae Externus pars Medialis lies ventrolateral to the m. AMES (Figs. 6, 7e, 8, 9d; [30, 33]). This peripheral origin and lateral insertion on the lower jaw suggests a potential homology with the m. AMES of lepidosaurs and turtles [47, 76, 97]. Internally the muscle includes a space that likely corresponds to a prominent aponeurosis (Fig. 7c; Additional file 1) [33]. It extends from near the origin, giving the muscle a pennate structure (Fig. 9b) and allows it to be further divided into an anterior (m. AMEMa) and posterior portion (m. AMEMb).

**Origin:** Three short aponeuroses: one originating from the postorbital process and two from the zygomatic process (Figs. 8a, 9d). This three-part origin is consistent with the description of van Gennip [33], rather than those by others [30, 38, 42], who only describe its origin from the processus zygomaticus (=temporalis).

**Path:** Both parts pass anterior ventrally lateral to the location of the m. Adductor Mandibulae Profundus and m. Pseudotermporalis profundis.

**Insertion:** A broad, fleshy insertion on the lateral surface of the lower jaw (Fig. 8c; [30, 33]).

**Function:** Contraction of this muscle rotates the lower jaw upwards. It is particularly active between the stationing and transport phases of feeding [27] as well as the jaw-closing phase of drinking [28].

#### m. Adductor Mandibulae Externus pars Profundus (m. AMEP)

The medial boundary of the m. AMEP has been reported to be difficult to separate from the lateral portion of the m. Adductor Mandibulae Posterior [33, 105]. However, in our dataset this division can be discerned on both sides.

**Origin:** The dorsolateral part of the anterolateral surface of the quadrate (Figs. 6a, 7f, 9e).

**Path:** Passes anteroventrally and converges anteriorly (Figs. 7f, 9e).

**Insertion:** On a small point on the dorsal surface of the lower jaw posterior to the coronoid process (Fig. 6c, d; [30, 33].

**Function:** Contraction of this muscle rotates the lower jaw upwards. Its in vivo activity has not been examined directly [27, 28].

#### m. Adductor Mandibulae Posterior (m. AMP)

As mentioned above, we find the m. AMP to be reasonably distinct from the m. AMEP (*contra* [33]). It is also clearly separated from the m. Pseudotemporalis Profundus [33, 105] in contrast to the arrangement in *Columba palumbus* [39, 105].

**Origin:** The ventral part of the anterolateral surface of the quadrate (Fig. 8a) ventro medial to the origin of the mAMEP.

**Path:** The muscle passes anteroventrally medial to the m. AMEP (Figs. 7g, 9f).

**Insertion:** A flat triangular surface on the dorsal surface of the mandible immediately anterior to the jaw joint (Fig. 8c, d, [30, 33]).

**Function:** Given its size and location, the m. AMP probably plays a role in stabilising the lower jaws during closure and possibly in jaw opening [38]. Its in vivo activity has not been examined directly [27, 28].

#### m. Pseudotemporalis (m. Pst)

The m. Pseudotemporalis is part of the internal jaw adductor complex that lies medial to the lateral-most extent of CN V (Fig. 6; [97]). Within Sauropsida (e.g., [47, 76, 79]), it is often divided into superficial and deep muscles with separate origins and insertions. This is also the case in *C. livia,* but not in *C*. *palumbus* in which the two muscles merge [39, 105].

#### m. Pseudotemporalis Superficialis (m.PstS)

The m. Pseudotemporalis superficialis is located posterior to the eye and medial to the path of CN V_2_. CN V_3_ passes beneath its main body and through its ventral aponeurosis [30, 42].

**Origin:** This muscle has a broad fleshy origin on the posterior wall of the orbit (Figs. 6a, 7a, 8a, 9g; [30, 33]).

**Path:** Anteroventrally and converging anteriorly (Figs. 7a, 9g). In the CT dataset, CN V_2_ passes ventral to the main body of the segmented muscle. This is where the aponeurosis would be and therefore the location of the nerve is consistent.

**Insertion:** A narrow point on the lower jaw via an aponeurosis (Fig. 6c, d; [30, 33]).

**Function:** Contraction of this muscle closes the jaws. Its in vivo activity has not been examined directly [27, 28].

#### m. Pseudotemporalis Profundus (m. PstP)

Variation in the size of this muscle (=m. quadratormandibularis in [105]) among columbiform birds has been linked to differences in beak length [33, 42]. It is situated medial to the pterygoid branch of CN V_3_ (sensu Bhattacharyya [30]) and m. AMP (Figs. 6, 7a, 9h; Additional file 1), whereas the main branch of CN V_3_ passes through it and into the Meckelian groove (Fig. 4).

**Origin:** A small region on the medialmost end of the medial process of the quadrate (Fig. 8a) medial to the mAMP.

**Path:** Anterolaterally (Fig. 9h). Close to its insertion, it bifurcates either side of CN V_3_, which continues into the Meckelian groove (Fig. 4).

**Insertion:** A broad area on the lateral, dorsal, and medial surfaces of the lower jaw anterior to the coronoid process; this insertion also includes the majority of the adductor fossa (8 c, d, 9 h; [33, 105]).

**Function:** Contraction of this muscle closes the jaws. It is particularly active during the grasping phase of feeding [27], as well as the jaw closing phase of drinking [28].

#### m. Pterygoideus (m. PT)

The m. pterygoideus is frequently the largest jaw muscle complex in amniotes, and is often divided into separate dorsal and ventral muscles that have distinct origins and insertions [47, 79].

#### m. Pterygoideus Dorsalis (m. PtDo)

**Fig. 10:**
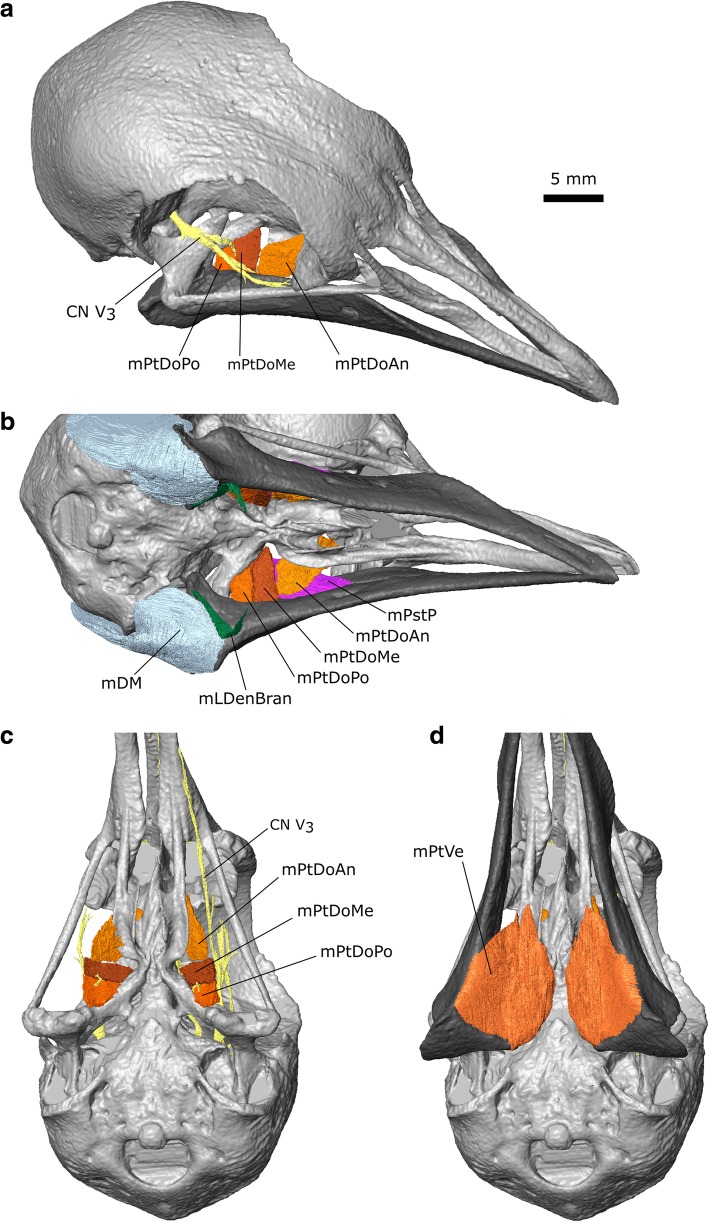
The pterygoideus muscles of *Columba livia*. **a** dorsolateral view. **b** ventrolateral view without the mPtVe in place. **c** ventral view without the lower jaw and mPtVe. **d** ventral view with the lower jaw and mPtVe

Previous descriptions have divided this muscle into anterior (m. PtDoAn) and posterior portions [30, 33]. We find that the posterior part (=caudalis of van Gennip [33]) can be further subdivided into posterior (m. PtDoPo) and medial parts (m. PtDoMe) due to a gap between the parallel muscle fibres through which passes the pterygoid branch of CN V_3_ (sensu Bhattacharyya [30], Figs. 7h, 9i, 10c; Additional file 1). The dorsal exit of this branch was figured by van Gennip ([33]: Fig. 17). The m. Pterygoideus Dorsalis of Bhattacharyya [30] seems to only include the posterior (m. PtDoPo) and medial parts (m. PtDoMe) with the anterior part left either undescribed or as part of the m. Pterygoideus ventralis.

**Origin:** The anterior part (=rostralis of van Gennip [33]) originates from the dorsolateral surface of the palatine whereas the medial and posterior parts originate from the lateral surface of the pterygoid (Figs. 8a, 9i, 10). We do not observe any part of the anterior part originating from the pterygoid (*contra* Bhattacharyya [30]).

**Path:** The anterior part passes posteroventrally past the insertion of the m. PstP, whereas the medialis and posterior have a more lateral inclination (Figs. 7h, 9i, 10abc).

**Insertion:** The anterior part inserts posteroventral to the insertion of the m. PstP, via an aponeurosis [33] whereas the posterior and medial parts insert on the medial surface of the lower jaw between the insertion of the m. PstP and the point of jaw articulation, and posterodorsal to the insertion of the m. PtDoAn (Fig. 10a, b, c; Additional file 1).

**Function:** Contraction of these muscles closes the lower jaw and provides a significant supportive role against torsion or long axis bending of the jaws.

#### m. Pterygoideus Ventralis (m. PtVe)

Like the m. PtDo, the m. Pterygoideus ventralis in *C*. *livia* is often subdivided but there has been little consensus on the exact boundaries between these divisions in Columbiformes [33, 40, 42]. In our dataset, a division into two parts is incomplete and the lateral portion (referred to as the “venter externus” by Burton [40]), is not as large as that present in columbiform birds that eat fruit such as *Didunculus* [25, 31, 40].

**Origin:** The anterior-most part of the muscle originates from the posterolateral end of the ventral surface of the palatine whereas the posterior-most part originates from the ventral surface of the pterygoid (Figs. 6a, 9j; [33]).

**Path:** Fanning out posterolaterally (Figs. 9j, 10d).

**Insertion:** On the medial surface and posterolateral corner of the lower jaw (Fig. 10d; [33]).

**Function:** Contraction of the m. PtVe is particularly important for closing the jaws at large gapes. It is especially active during the grasping phase of feeding [27], but is also continuously active during drinking, with slightly greater activity during the jaw closing phase of drinking [28].

#### m. Protractor Pterygoidei et Quadrati (m. PPQ)

This muscle lies between the braincase and pterygoid-quadrate bar (Figs. 7, 9k) medial to CN V (Figs. 6, 7). As in lepidosaurs [47], some birds have two protractor muscles: one between the sphenoid and the pterygoid bones, and another from the sphenoid to the quadrate [97]. However, in some other birds the two protractors are inseparable at their origin [58]. Descriptions of this muscle in columbiform birds may refer to two separate muscles [42], but the majority report that there is no separation (e.g., [25, 30, 33, 38]). Our results are consistent with the latter interpretation although the origin has two distinct heads.

**Origin:** From the posterior surface of the orbit (Fig. 8a).

**Path:** The muscle divides into two heads. The posterior, dorsal-most head continues anterolaterally, whereas the anterior, ventral-most head passes more laterally (Fig. 9k).

**Insertion**: The dorsal-most head inserts on the dorsal edge and posterior surface of the anteromedial process of the quadrate whereas the ventral-most head inserts onto the posterior surface of the pterygoid (Fig. 8b).

**Function:** Contraction of this muscle facilitates protraction of the pterygo-quadrate-maxillopalatine complex and opening of the upper jaw [27, 31]. It is active during both the grasping and stationing phases of feeding [27] as well as the jaw closing phase of drinking [28]. The frequent activity of the m. PPQ during feeding and the absence of variation associated with pellet size suggest that it has an important role in “unlocking” the kinetic mechanism [27].

#### m. Depressor Mandibulae (m. DM)

**Fig. 11:**
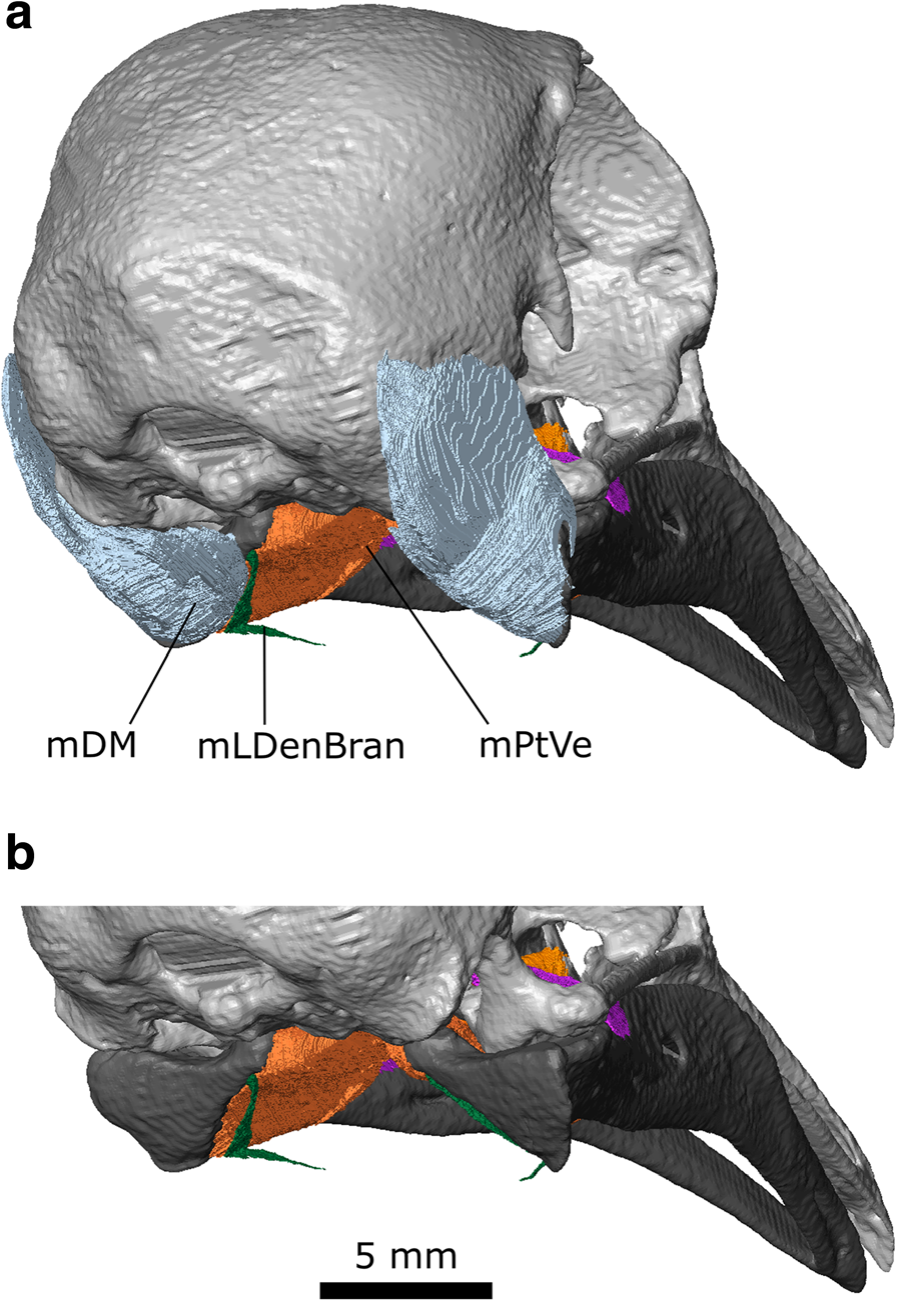
The depressor mandibulae of *Columba livia* in posterior view

This muscle in *Columba* has previous been divided in two [30] and three parts [33] but in our CT datasets we cannot distinguish any complete divisions (Figs. 9l and 11).

**Origin:** A broad area on the posterolateral surface of the squamosal (Figs. 6a, 10, 11) as well as the posterior and lateral surface of L2 (Fig. 5).

**Path:** It passes ventrolaterally and converges towards its insertion (Figs. 9l and 11a).

**Insertion:** On the lower jaw over a broad continuous area including its dorsolateral corner, the flat posterior surface of the lower jaw, and the posterior end of the medial surface (Figs. 6c, d and 11).

**Function:** This muscle primarily rotates the lower jaw; however, this muscle can also be involved in protraction of the upper jaw due to the mobility of the quadrate, postorbital ligament and action of other muscles such as the m. Protractor Pterygoidei et Quadrati [27, 95]. It is most active just prior to the gape, stationing, and intraoral transport phase of feeding [27], as well as the jaw opening phase of drinking [28]. Its duration of activity is extended when dealing with particularly large food items [27].

#### Throat and tongue muscles

Previous descriptions of throat muscles in columbiform birds include those of Burk [37], Lubosch [106], Rawal [36], Burton [40], Bhattacharyya [30], and Zweers [32]. Further useful descriptions of throat anatomy in birds include Engels [107] Harvey et al. [108], McLelland [109], George and Berger [110], Zusi and Storrer [111], and Huang et al. [112]. Their sites of origin and insertion, as well as developmental history (based on the chicken *Gallus gallus*), can be used to divide the throat muscles into three groups: glossal, suprahyoid, and infrahyoid [112]. Previous descriptions have used various synonyms for many of these muscles (Additional file 3). Here we mainly use the terminology of Bhattacharyya [30], and Huang et al. [112].

#### Suprahyoid muscles

These muscles extend from the lower jaw to the hyobrachial skeleton [112]. They are homologous to the suprahyoid muscles of mammals [112]. They do not originate from somites and may derive from preoptic paraxial mesoderm [112, 113].

#### m. Mylohyoid (m. Mylo)

**Fig. 12:**
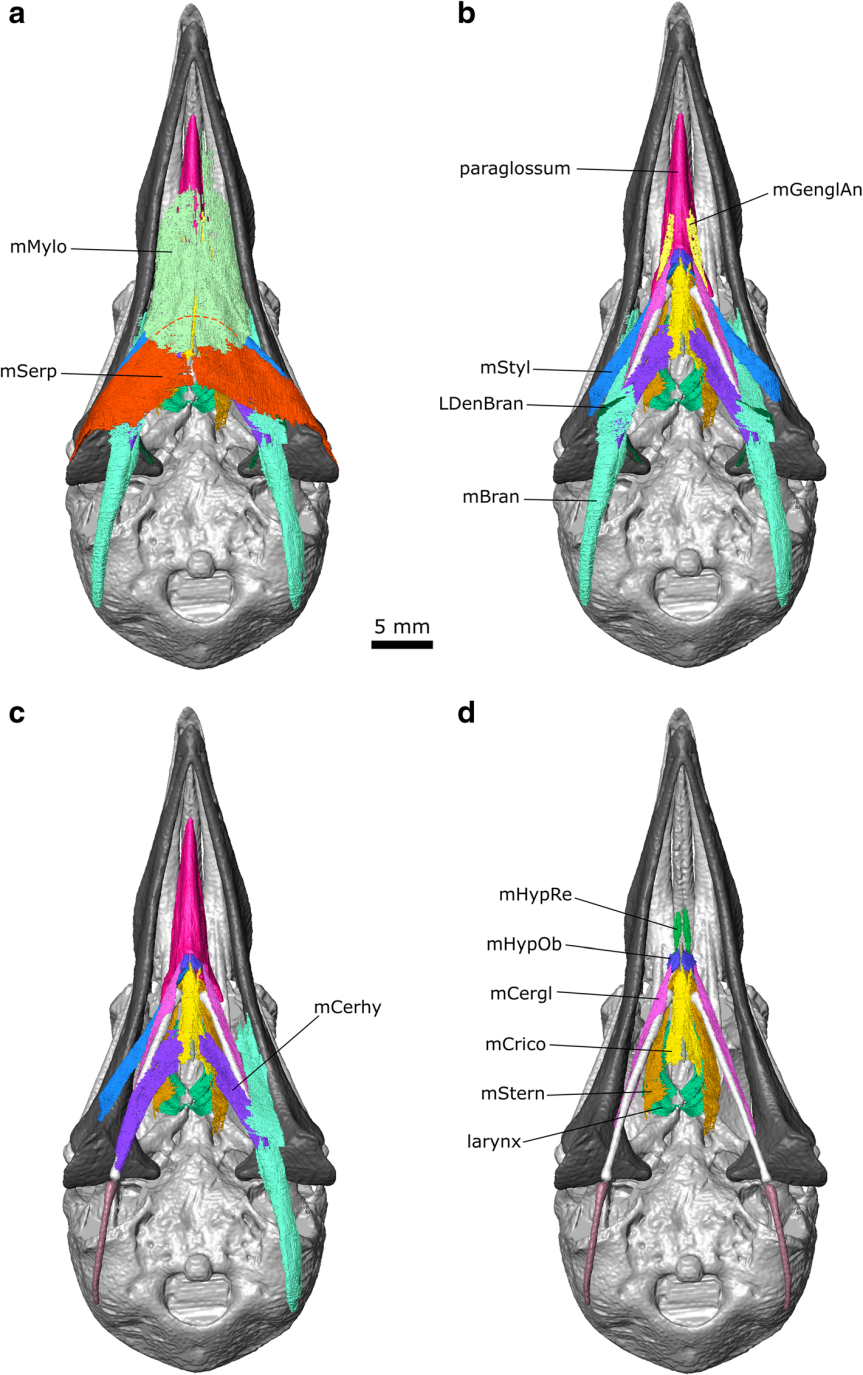
The throat muscles of *Columba livia* in ventral view. **a** to **d** represent increasingly deep dissections

This is a broad flat muscle (=m. Intermandibularis ventralis of Rawal [36] and = m. Intermandibularis ventralis rostralis of Zweers [32]) composed of parallel fibres, which forms the floor of the mouth. It is innervated by CN V_3_ [107] (Engels 1938). In *Columba* the m. Mylo was described as “delicate” by Burk [37] and “well developed” by both Rawal [36] and Bhattacharyya [30]. In CT datasets 2 and 3, it appears to be very thin and in parts almost discontinuous. Zweers [32] divided the muscle into three contiguous parts (rostral, middle, and caudal) based on changes in thickness and fibre direction. In our dataset the three parts are not obvious and the muscles appear to be very thin (Fig. 12). An absence of muscle along the posterior midline probably indicates the location of the midline raphe [32]. Posteriorly it is difficult to separate the m. Mylo from the m. Serpihyoideus, which it underlaps medially (Fig. 12).

**Origin:** From the lingual edge of the dorsal ridge of the lower jaws (Fig. 12).

**Path:** It passes ventrally and medially (Fig. 12).

**Insertion:** Into the median raphe (“medial strand” of Zweers [32]) at the midline of the anterior end of the throat (Fig. 12; [30, 32, 37]).

**Function:** Contraction of this muscle raises the floor of the mouth and reduces the volume of space within the oral cavity [30].

#### m. Serpihyoideus (m. Serp)

This muscle is spoon-shaped and composed of parallel fibres [30, 32]. Again an absence of muscle along the midline (Fig. 12) likely indicates the presence of the midline raphe [32]. The m. Serpihyoideus lies anterior to, and continuous with, the m. Intermandibularis ventralis of Zweers [32] which is not described here.

**Origin:** On the posterodorsal surface of the lower jaw just posterior to the articular (Fig. 6d), posterior and lateral to the origin of the m. Stylohyoidues, and anterior to the insertion of the m. DM [30, 32].

**Path:** It passes anteromedially and superficial to the location of the urohyale (Fig. 12).

**Insertion:** Inserts along the midline (Fig. 12) where a raphe would be [32], its anteromedial corner being overlapped by the m. Mylo [32].

**Function:** This muscle serves to support the tongue and assist with raising the floor of the mouth [30].

#### m. Stylohyoideus (m. Styl)

This is a slender, strap-like muscle that is composed of parallel fibres, which lies deep to m. Serp and m. Mylo [30, 33]. In the CT dataset the m. Styl and m. Serp are difficult to separate from one another along the surface of the lower jaw.

**Origin:** From the posterodorsal region of the lower jaw, just anterior and slightly ventral to the origin of the m. Serpihyodeus (Fig. 12b; [30, 32].

**Fig. 13:**
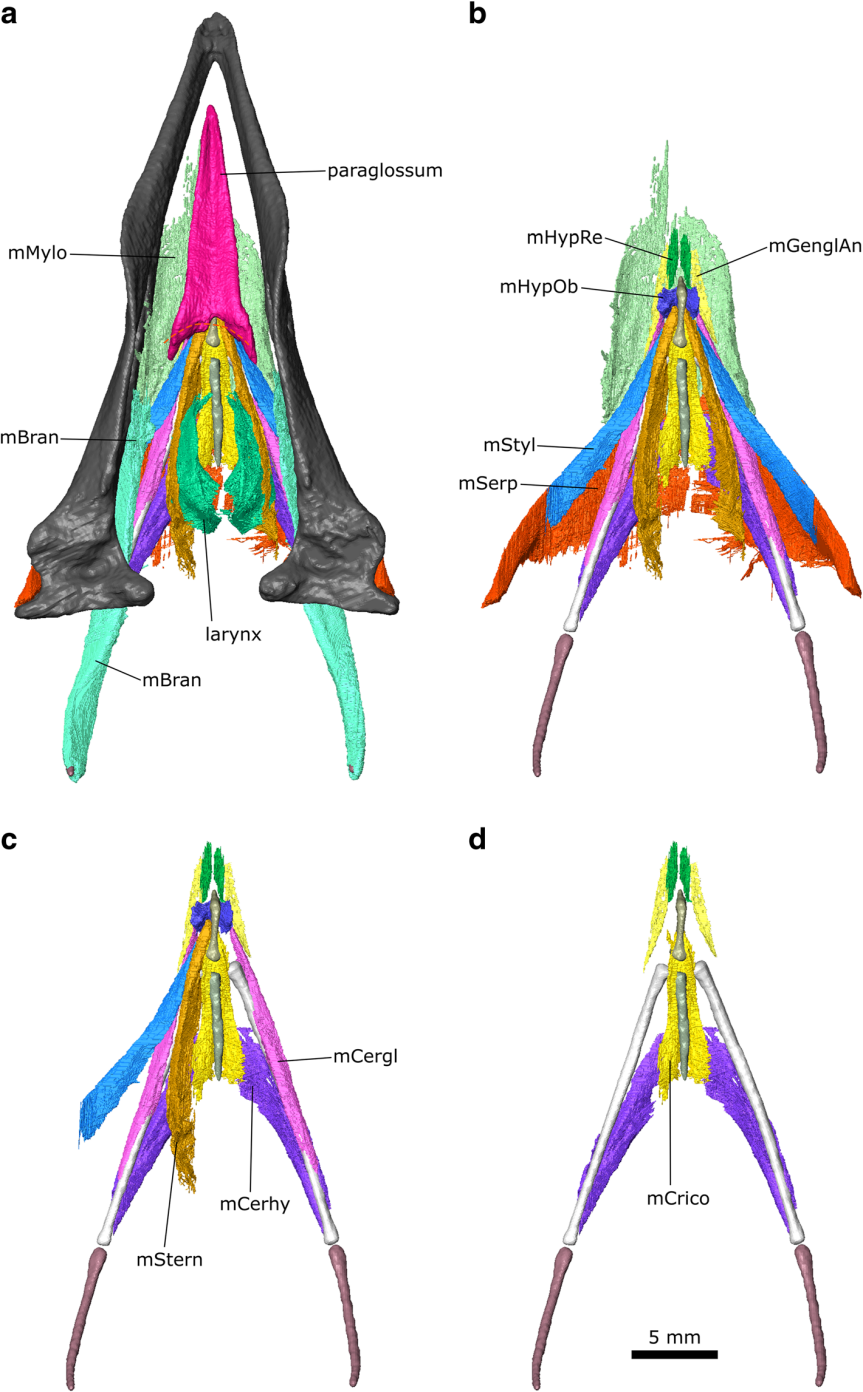
The throat muscles of *Columba livia* in dorsal view. **a** to **d** represent increasingly deep dissections

**Path:** It passes anteromedially deep to the m. Mylo and m. Serp (Figs. 12, 13).

**Fig. 14:**
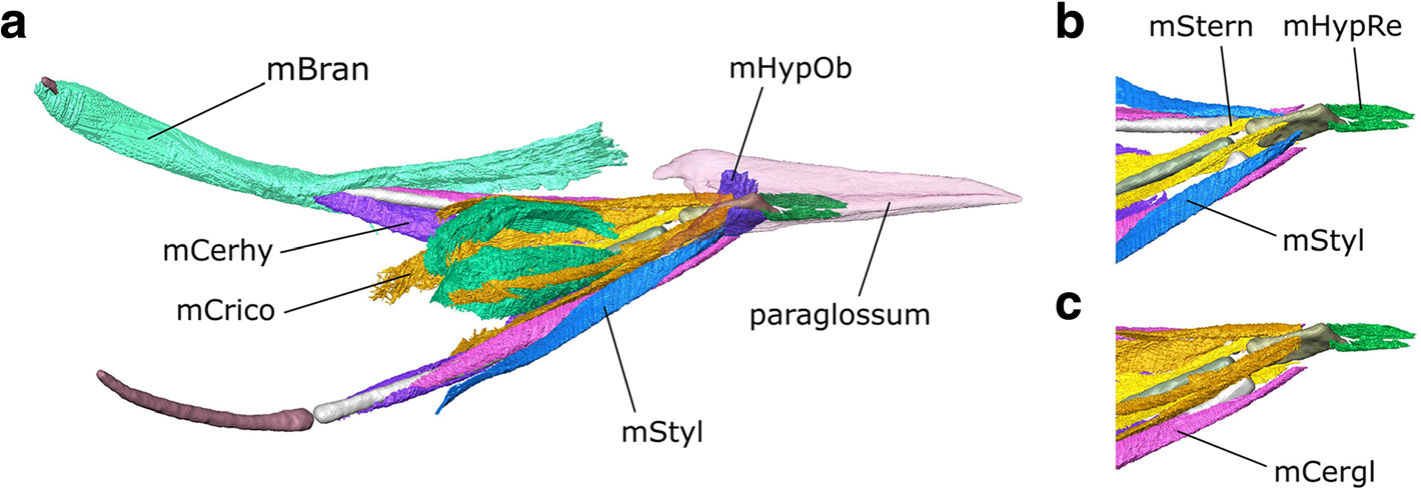
The throat muscles of *Columba livia* in lateral view. **b** and **c** show only the anterior portion with the paraglossum absent

**Insertion:** On the lateral surface of the basihyale close to the base of the paraglossum (Fig. 14b).

**Function:** Contraction of this muscle results in retraction of the hyoid apparatus.

#### m. Branchiomandibularis (m. Bran)

This is a long muscle (=m. Branchiohyoideus of Li and Clarke [68]) that extends from the lower jaw to the cornu branchiale [30, 32]. There are two heads near to the point of origin that have been used as a basis for dividing the muscle into two parts [30, 32], but these separate heads are not obvious in our CT datasets.

**Origin:** From the lingual surface of the central part of the lower jaw (Fig. 12c) close to where the m. Styl passes ventral to the m. Mylo.

**Path:** It passes posteroventrally (in contrast to many of the other muscles in the throat) and passes deep to the m. Mylo, m. Serp, and m. Styl.

**Insertion:** The medial and dorsolateral surfaces of the epibranchial (Figs. 12, 14; [30, 32].

**Function:** Contraction of this muscle results in protraction of the hyoid apparatus. Greater development of this muscle in the columbiform birds *Ducula* and *Treron* supposedly allows greater tongue prehension for eating fruit [25].

#### m. Ceratohyoidues (m. Cerhy)

This is a strap-like muscle with parallel fibres [30, 32] located between the ceratobranchial and the posterior half of the urohyale. Although this contrasts with the more anterior location of the m. Cerhy in 10-day old chick embryos ([112]: muscle 9), the location in adult chickens extends more posteriorly ([109]: muscle f, see also [94]) and thus more closely resembles the arrangement observed in the pigeon, consistent with the inferred homology followed here.

**Origin:** The medial surface of the posterior ends of the ceratobranchial (Figs. 12b and 13b).

**Path:** It passes anteromedially [30, 32].

**Insertion:** The median raphe near the posterior end of the urohyale (Figs. 12b and 13b; [30, 32]).

**Function:** Contraction of this muscle would pull the cornu branchiale toward the midline or resist the m. Bran separating them.

#### Glossal muscles

These muscles, attached to the paraglossum, are homologous to the mammalian extrinsic lingual muscles and develop from somites 2–6 [112].

#### m. Genioglossus Anterior (m. GenglAn)

This is a thin muscle that extends along the floor of the mouth [30, 32]. Along with the m. GenglPo, it was referred to as the m. Sternomandibularis by Lubosch [106] and the m. Geniopharyngealis by [32].

**Origin:** Reportedly from near the symphysis of the lower jaw [32], but not clearly visible in CT datasets 2 or 3.

**Path:** Posteriorly parallel to the midline (Fig. 12b).

**Insertion:** On the floor of the mouth [32] near to the anterior part of the cricoid cartilage (Fig. 12b; [30]). In the CT dataset some of the anterior part is visible, particularly on the right side.

**Function:** Contraction of this muscle may have pulled the hyoid apparatus forwards. Greater development of this muscle in *Ducula* and *Treron* supposedly allows greater tongue prehension [25].

#### m. Genioglossus Posterior (m. GenglPo)

The posterior part is not clearly visible in any of our CT datasets [30], but it reportedly originates from the floor of the mouth on either side of the anterior end of the trachea [32], passes posteriorly parallel to the midline [30] and inserts near the posterior part of the cricoid cartilage [30].

#### m. Hypoglossus Rectus (m. HypRe)

This is a relatively small muscle (= m. Hypoglossus in Anterior of Zweers [32]) located at the anterior end of the hyoid apparatus that extends parallel to the paraglossum [30, 32].

**Origin:** From the ventral end of the paraglossum (Fig. 14a) near to its articulation with the basihyale and the insertion of the m. Ceratoglosssus (Fig. 12d). In chickens, the origin of this muscle may also involve the basihyale [109, 112].

**Path:** Anteriorly parallel to the midline (Fig. 12d).

**Insertion:** The anterior surface of the entoglossum and posterior end of the paraglossum (Figs. 12d, 14).

**Function:** Contraction of this muscle results in flexion of the paraglossum.

#### m. Hypoglossus Obliquus (m. HypOb)

This is a small muscle located at the anterior end of the hyoid apparatus and it extends perpendicular to the long-axis of the paraglossum [30, 32].

**Origin:** It originates from the ventral edge of the anterolateral surface of the basihyale (Figs. 13b, 14a).

**Path:** The muscle passes dorsolaterally (Fig. 14a).

**Insertion:** The posteroventral surface of the caudal horn of the paraglossum (Fig. 14a; [30, 32]).

**Function:** Contraction of this muscle results in depression of the paraglossum [109].

#### m. Ceratoglossus (m. Cergl)

This is a narrow strap like-muscle [30, 32].

**Origin:** This muscle originates from the dorsolateral surface of the posterior end of the ceratobranchiale (Fig. 13d; [30, 32]).

**Path:** This muscle passes anteroventrally along the lateral surface of the ceratobranchiale (Fig. 13c) deep to the m. Bran.

**Insertion:** The posterolateral end of the paraglossum via a tendon that passes ventral and lateral to the anterior-most end of the m. Styl (Fig. 13d; [30, 32]).

**Function:** Contraction of this muscle results in ventral and ventrolateral movement of the paraglossum [109].

#### Infrahyoid muscles

These muscles extend from the larynx, tracheal cartilage, clavicle, and sternum to the hyobrachial skeleton and develop from somites 2–6 [112].

#### m. Cricohyoideus (m. Crico)

This muscle (= m. Thyrohyoideus of Bhattacharyya [30]) lies close to the midline. It originates on the ventral surface of the anterior end of the basihyal and inserts around the cricoid cartilage [30, 32].

**Origin:** This muscle originates from the posterior of the throat [112].

**Path:** It passes anteriorly parallel to the midline [30].

**Insertion:** Posterior end of the paraglossum and dorsal surface of the basihyale, dorsal to the insertion of the m. Styl (Fig. 14c).

**Function:** Contraction of this muscle retracts the hyoid apparatus.

#### m. Sternohyoides (m. Stern)

This is a strap-like muscle (= m. Claviculohyoideus of Zweers [32]).

**Origin:** This muscle originates from the sternum [112].

**Path:** It passes anteriorly lateral to the trachea and m. Crico [30].

**Insertion:** Posterior end of the paraglossum and dorsal surface of the basihyale (Fig. 14c), dorsal to the insertion of the m. Styl.

**Function:** Retraction of this muscle retracts the hyoid apparatus.

### Eye muscles

**Fig. 15:**
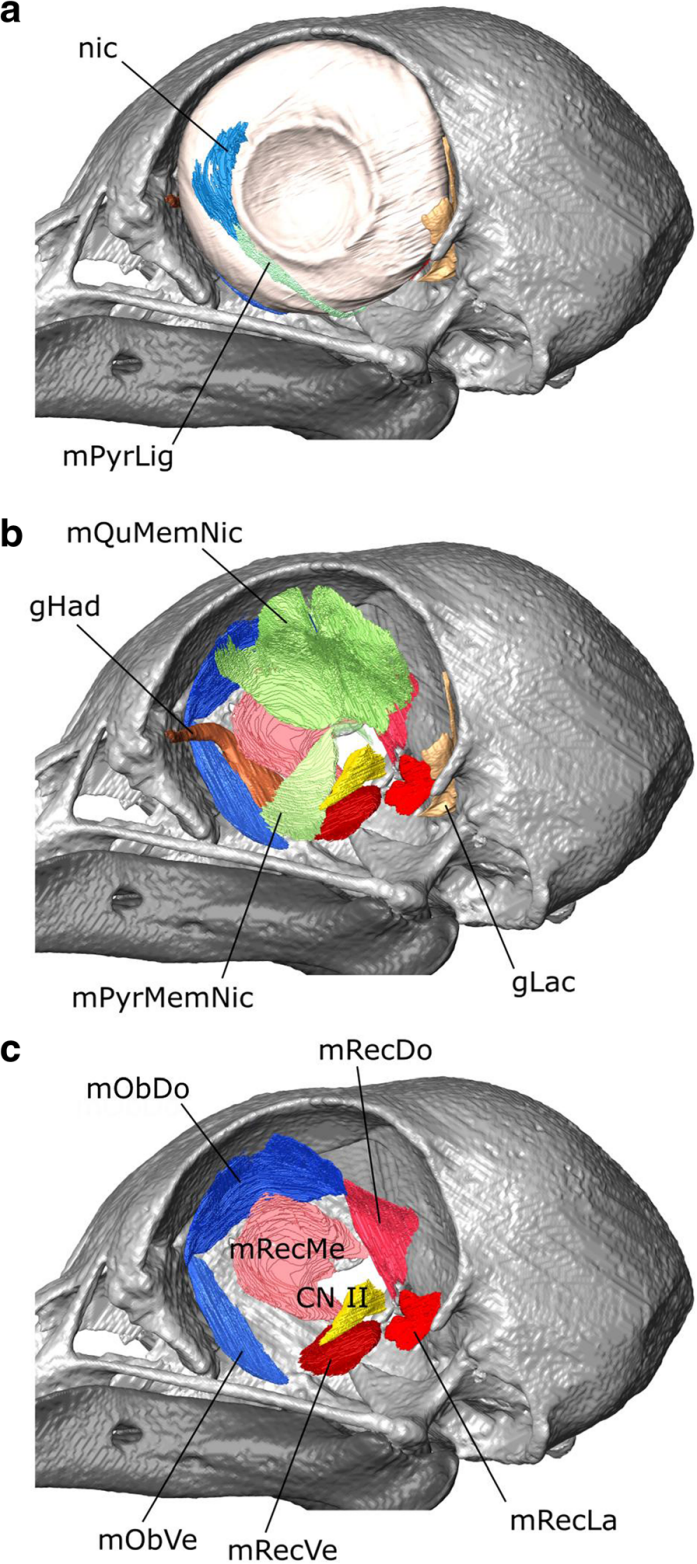
The eye muscles of *Columba livia* in lateral view. **a** to **c** represent increasingly deep dissections. **a** the nictitating membrane and associated ligament, **b** the intrinsic and extrinsic muscles, **c** the extrinsic muscles only

**Fig. 16:**
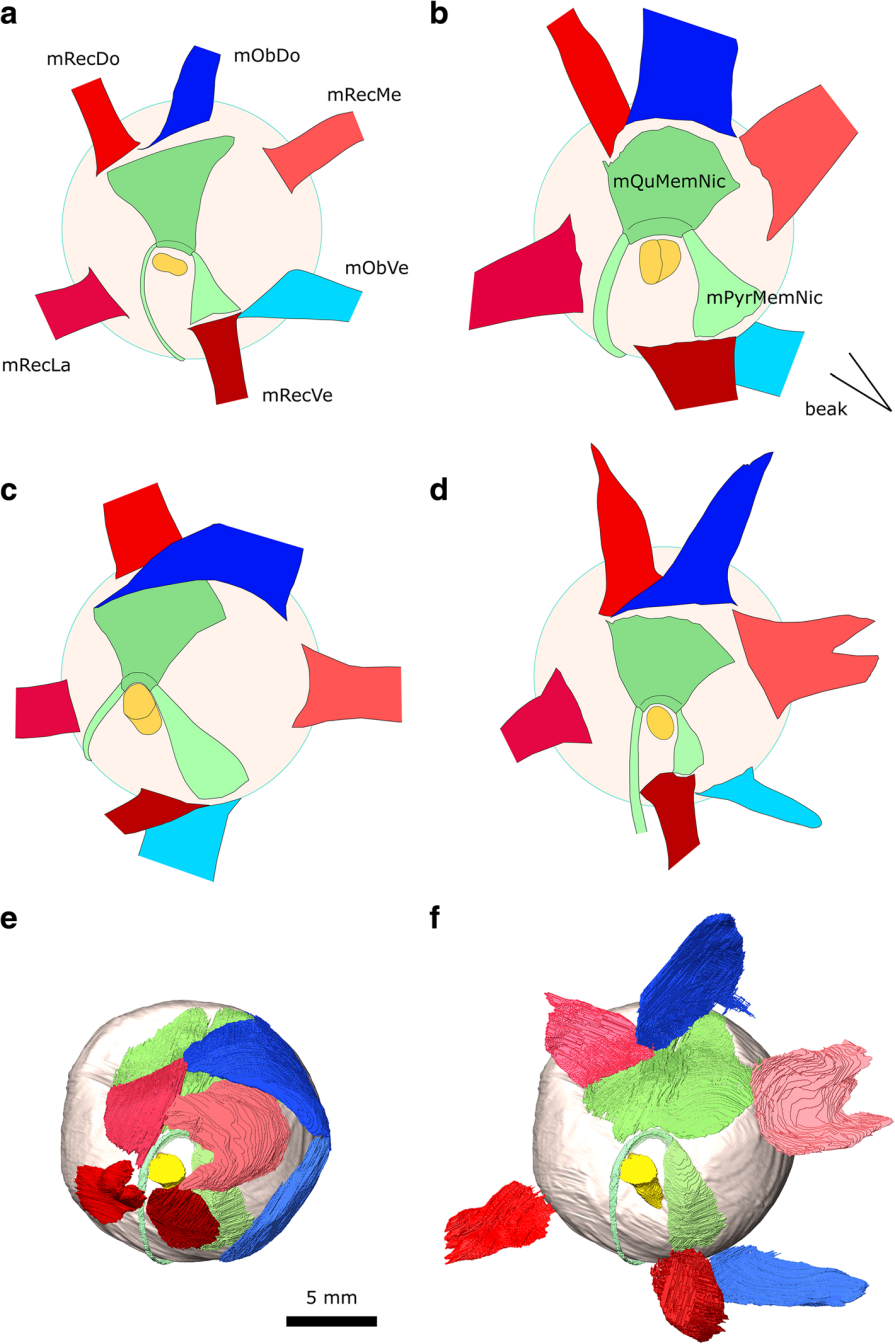
Eye muscle arrangement in medial view of the left eye in **a** *Passer domesticus*, sparrow, (redrawn from Slonaker [99] and reversed for comparison) **b** *Meleagris gallopavo*, turkey (redrawn from Harvey et al. [108]) and reversed for comparison) **c** *Eudromia elegans*, elegant crested tinamou (redrawn from Elzanowski [115]) **d** *Columba livia*, the rock dove (redrawn from Chard and Gundlach [102]) **f** *Columba livia* digital dissection **e** *Columba livia* digital dissection with the rectus and obliquus muscles folded outwards

In birds there are eight eye muscles (six extrinsic and two intrinsic) and two or three eyelid muscles [37, 99, 110, 114]. The extrinsic muscles comprise four strap-like (or petal-like) rectus muscles and two elongate oblique muscles, whereas the intrinsic muscles are associated with the nictitating membrane (Figs. 15a, 16; [114]). The eye muscles of the pigeon have been described by Chard and Gunlach [102] (Fig. 16d) and more briefly by Burk [37] and Knox and Donaldson [116]. The intrinsic and eyelid muscles were summarised by George and Berger [110]. Other useful descriptions of eye muscles in birds include the comprehensive study of the house sparrow (*Passer domesticus*) Slonaker [99] (Fig. 16a), images of eye muscle anatomy in the domestic turkey (*Meleagris gallopavo*) Harvey et al. [108] (Fig. 16b), a description of the oblique and nictitating muscles in the ostrich (*Struthio camelus*) Webb [100], and a detailed investigation of the eye muscles in Tinamiformes (Fig. 16c; [115]). These descriptions permit a preliminary cross taxon comparison (Table 4).

**Table 4:** Morphological traits among the eye muscles of birds. Size comparisons for the rectus muscles are mainly based on their size at the point of insertion

	Sparrow	Turkey	Tinamou	Pigeon	Pigeon
Fig. 16	a	b	c	d	f
Ref	Slonaker 1918	Harvey et al. 1968	Elzanowski 1987	Chard and Gundlach 1937	Our digital segmentation
	[99]	[108]	[115]	[102]	na
size of the mObDo relative to the mRecDo	similar	twice as wide	twice as wide	wider	wider
size of m. RecLa vs mRecMe	larger	smaller	smaller	smaller	smaller
location of the insertion of the mRecVe relative to the insertion of the mPyrMemNic	level	lateral	far lateral	lateral	lateral
extent of overlap by the mRecVe of the origin of the mPyrMedNic	~ 100%	~ 40%	< 5%	~ 40%	~ 40%
size of the mRecDo vs mRecVe	similar	smaller	larger	larger	larger
location of the insertion of the mObVe relative to the insertion of the mRecVe	far lateral	lateral	lateral	far lateral	lateral
extent of overlap by the mObVe of the origin of the mRecVe	< 5%	~ 70%	~ 70%	< 5%	~ 50%
proximity of the insertion of the mRecMe to the mObDo	distant	very close	close	very close	distant?
Location of the insertion of the mRecDo relative to that of the mQuMemNic	aligned at lateral edges	aligned at lateral edges	aligned at medial edges	aligned at lateral edges	aligned at lateral edges
mRecMe > mRecLa	no	no?	yes	yes	yes
mRecMe > mRecDo	no	yes	no	yes	yes
mRecMe > mRecVe	no	no?	yes	yes	yes
mRecDo > mRecVe	no	no	yes	yes	yes
mRecDo > mRecLa	no	no	yes	no	no
mRecLa > mRecVe	slightly	slightly	slightly	slightly	slightly
m. Palpebrae Obliquus (m. LevPalOb)	absent	Not reported	present	Not reported	absent

#### m. Quadratus Membrane Nictitans (m. QuMemNi)

As in all birds previously described, the m. Quadratus Membrane Nictitans is the larger of the two muscles associated with the nictitating membrane [58, 99, 100, 102]. It is innervated by CN VI [114].

**Origin:** A wide region of the dorsal part of the eye (Figs. 15a, 16f). It appears to be wider than in previous descriptions for the pigeon (Fig. 16d; [102]) which might reflect the difficulties in interpretations of where muscle ends and tendon begins.

**Path:** It covers a significant part of the medial surface of the eyeball converging towards CN II. In medial view it is partly obscured by the m. Rectus Dorsalis, m. Rectus Medialis, and m. Obliquus Dorsalis (Fig. 16e).

**Insertion:** As visible in the third CT dataset (Fig. 16f), it forms a sling [100, 114] around the tendon of the m. pyramidalis membrane nictitans just dorsal to CN II (Fig. 16e).

**Function:** Contraction draws the nictitating membrane posteriorly over the cornea of the eye, whereas relaxation allows the nictitating membrane to return to its resting position along the anterior edge of the eye [99, 100, 110, 114].

#### m. Pyramidalis Membrane Nictitans (m. PyrMemNi)

The m. Pyramidalis Membrane Nictitans is the smaller of the two muscles associated with the nictitating membrane [37, 58, 99, 100, 102]. The tendinous end is visible in the third CT dataset. We find the muscle belli to be much larger than figured by Chard and Gundlach [102].

**Origin:** The m. PyrMemNi has previously been described as arising from the ventral surface of the eye [102], but our segmentation suggests that the origination is anteroventral rather than strictly ventral (Fig. 16f). It is innervated by CN VI [114]. In correspondence with previous descriptions of the pigeon [102], about 50% of the origin is overlapped by insertion of the m. Rectus Ventralis [102]. This organisation contrasts with that of the sparrow (Fig. 16a; [115]) where the origin is entirely overlapped and in the tinamou where there is almost no overlap (Fig. 16c; [115]).

**Path:** The m. PyrMemNi has a complex course that first passes dorsally and transitions into a narrow tendon (Figs. 15a, 16f) that curves posteriorly through a sling formed by the m. QuMemNi, around CN II, and passes posteroventrally around the eye before heading anterodorsally [102, 110].

**Insertion:** On the posteroventral edge of the membrane nictitans (Fig. 15a).

**Function:** As for the m. QuMemNi, contraction draws the nictitating membrane posteriorly over the cornea of the eye, whereas relaxation allows the nictitating membrane to return to its resting position along the anterior edge of the eye [99, 110]. The involvement of both muscles, with the sling and tendon mechanism, maintains a consistent direction of pull on the pyramidalis tendon regardless of the orientation of the eye [99, 100, 110, 114].

#### m. Rectus Lateralis (m. RecLa)

The m. Rectus Lateralis (=external rectus of Chard and Gunlach [102]) lies in the posteroventral corner of the orbit and is innervated by abducens nerve (CN VI).

**Origin:** Like the other rectus muscles, the m. Rec La originates from a thickened periorbital tissue surrounding the optic nerve (Fig. 15c; [102, 110, 116]). The muscle origin involves two heads of equal size (Fig. 16e).

**Path:** It follows the medial surface of the eye (Fig. 15e).

**Insertion:** The posteroventral surface of the eye (Fig. 15e). The insertion is similar in size to that of the house sparrow (Fig. 16a; [99]) and tinamou (Fig. 16c; [115]).

**Function:** Contraction of the m. RecLa alone moves the eye posteroventrally [116].

#### m. Rectus Medialis (m. RecMe)

The m. Rectus Medialis (=internal rectus of Chard and Gunlach [102]) is a relatively large, flat muscle.

**Origin:** The anteromedial edge of a thickened periorbital tissue surrounding CN II (Fig. 15c) close to the interorbital septum. The muscle origin involves two heads (Fig. 16e). The dorsal head may be larger than that of the m. Rectus Ventralis in contrast to previous descriptions (Fig. 16d; [102]). It is innervated by CN III (Additional file 1).

**Path:** The m. RecMed is situated in the centre of the orbit close to its medial wall, dorsal to the path of CN III and superficial (lateral) to the paths of the Harderian gland and CN V_1_. It follows the medial surface of the eye outside the m. QuMemNi and m. PyrMemNi (Fig. 16e).

**Insertion:** The anterodorsal surface of the eye near the m. Obliquus Dorsalis (Fig. 16e; [116]). The insertion is relatively large, similar in size to that of the turkey (Fig. 16b; [108]) and significantly larger than that of the house sparrow (Fig. 16a; [99]).

**Function:** Contraction of the m. RecMe alone moves the eye anterodorsally [116].

#### m. Rectus Ventralis (m. RecVe)

In *Columba livia*, the m. Rectus Ventralis (=inferior rectus of Chard and Gunlach [102]) is the smallest of the four rectus muscles [116].

**Origin:** The anteroventral edge of the thickened periorbital tissue surrounding CN II (Fig. 15c).

**Path:** Around the anteroventral surface of the eye overlapping the origin of the m. PyrMemNi (Fig. 16e).

**Insertion:** The anteroventral surface of the eye (Fig. 16e; [116]). The insertion is relatively similar in size to that of the house sparrow (Fig. 16a; [99]) and tinamou (Fig. 16c; [115]) but much smaller than that of the turkey [108].

**Function:** Contraction of the m. RecVe moves the eye anteroventrally [116].

#### m. Rectus Dorsalis (m. RecDo)

The m. rectus dorsalis (=superior rectus of Chard and Gunlach [102]) has been described as the largest of the four rectus muscles in *Columba* [116]. However, as elsewhere [102], we find it to be smaller than the m. Rec Med (Fig. 16 e, f). On the right it was difficult to separate the m. RecDo from the m. Quadratus Membrane Nictitantis. Similarly, the m. RecDo could not be clearly identified in a digital dissection of common buzzard (*Buteo buteo*), suggesting that this muscle is generally difficult to distinguish [58].

**Origin:** From the posterodorsal edge of the thickened periorbital tissue surrounding CN II (Fig. 15c).

**Path:** It lies close the posterior wall of the orbit; it is superficial (lateral) to CN IV, but deep (medial) to CN V_1_ (Fig. 16e).

**Insertion:** The posterodorsal surface of the eye (Fig. 16e; [116]). It is similar in size to that of the tinamou (Fig. 16c; [115]) rather than the house sparrow (Fig. 16a; [99]).

**Function:** Contraction of the m. RecDo alone causes the eye to move posterodorsally [116].

#### m. Obliquus Dorsalis (m. OblDo)

The m. Obliquus Dorsalis (=superior oblique of Chard and Gunlach [102] and Knox and Donaldson [116]) is one of two oblique muscles. On the right side it was difficult to distinguish from the m. Quadrates Membrane Nicititantis. It is the only muscle innervated by the trochlear nerve (CN IV).

**Origin:** The anterior wall of the orbit [102, 116]. In our segmentation, its origin is located just anterolateral to that of the m. Obliquus Ventralis on the anterior wall of the orbit (Fig. 13c).

**Path:** It passes medial to the course of CN V_1_, the m. RecMe, and the m. Quadrates Membrane Nicititantis (Fig. 16e; Additional file 1).

**Insertion:** The dorsomedial edge of the eye. The posterior part of the insertion is less extensive than might be expected given previous descriptions [102, 116] but it does lie medial to that of the m. RecDo (Fig. 16e). It is notably larger than in the house sparrow (Fig. 16a; [99]).

**Function:** Rotates the eye anterodorsally.

#### m. Obliquus Ventralis (m. OblVe)

The m. Obliquus Ventralis (inferior oblique of Chard and Gunlach [102] and Knox and Donaldson [116]) is a thin, strap-like muscle. It is innervated by CN III (Fig. 13c; Supp Mat 1).

**Origin:** The anterodorsal wall of the orbit [116] posteromedial to the m. OblDo (Fig. 16e).

**Path:** It passes medial to the Harderian gland and m. PyrMemNi (Fig. 16e).

**Insertion:** The ventral surface of the eye lateral to the m. RecVe (Fig. 16e). It appears to be larger than found in previous descriptions of the pigeon [102].

**Function:** Rotates the eye anteroventrally.

#### m. Levator Palpebrae Dorsalis (m. LevPalDo)

This muscle (=m. orbicularis palpebrarum superior of George and Berger [110]) is a thin sheet-like muscle located in the anterodorsal corner of the eye (Fig. 2a).

**Origin:** From an aponeurosis that arises from the dorsal or anterodorsal margin of the orbital boundary [110].

**Path:** It passes over the anterodorsal surface of the eye (Fig. 2a).

**Insertion:** Inserts on the dorsal eyelid (Fig. 2a).

**Function:** It opens the eyelid (Fig. 2a).

#### m. Levator Palpebrae Ventralis (m. LevPalVe)

This muscle (=m. orbicularis palpebrarum inferior of [110]) is a wide thin sheet-like muscle (Fig. 2a). It is innervated by CN3 [114].

**Origin:** A strong aponeurosis from the posteroventral corner of the orbit [110] ventral to the m. Rec Lat but dorsal to the m. PPQ and m. PstS (Fig. 8a; Additional file 1).

**Path:** It passes laterally between the m. RecLat and m. PPQ and around the ventrolateral edge of the eye.

**Insertion:** Inserts on the anterior surface of the ventral eyelid (Fig. 2a).

**Function:** It opens the eyelid. It may also serve to anchor the lid and prevent it being pulled forwards with the nictitating membrane when the nictitating muscles relax [114].

#### m. Levator Palpebrae Obliquus (m. LevPalOb)

This muscle, known to occur in the tinamou (=m. orbicularis palpebrarum of [115]), cannot be seen in any of our CT datasets and may be absent in *Columba*.

### Neck muscles

The homology of diapsid neck muscles has been generally established [117, 118] and detailed descriptions of bird muscles are available [110]. Burk [37] includes specific descriptions of *Columba livia*. Other useful descriptions of neck muscles in birds include those of the pied-billed grebe (*Podilymbus podiceps*) [111], the domestic turkey (*Meleagris gallopavo*) ([108] plates 7 to 13), and the common buzzard (*Buteo buteo*) [58].

#### m. Complexus (m. Cpx)

The m. Complexus of birds (e.g., [37, 108, 111]) is probably homologous with the M. Longissimus Cervicocapitis pars m. Articulo-parietalis of lepidosaurians and the m. Transversospinalis Capitis of crocodylians due to their similar points of origin and insertion [117]. It is the most superficial neck muscle in dorsal view [58, 110].

**Origin:** From the lateral surfaces of the cervical vertebrae posterior to the axis [37, 58, 110, 111, 119].

**Fig. 17:**
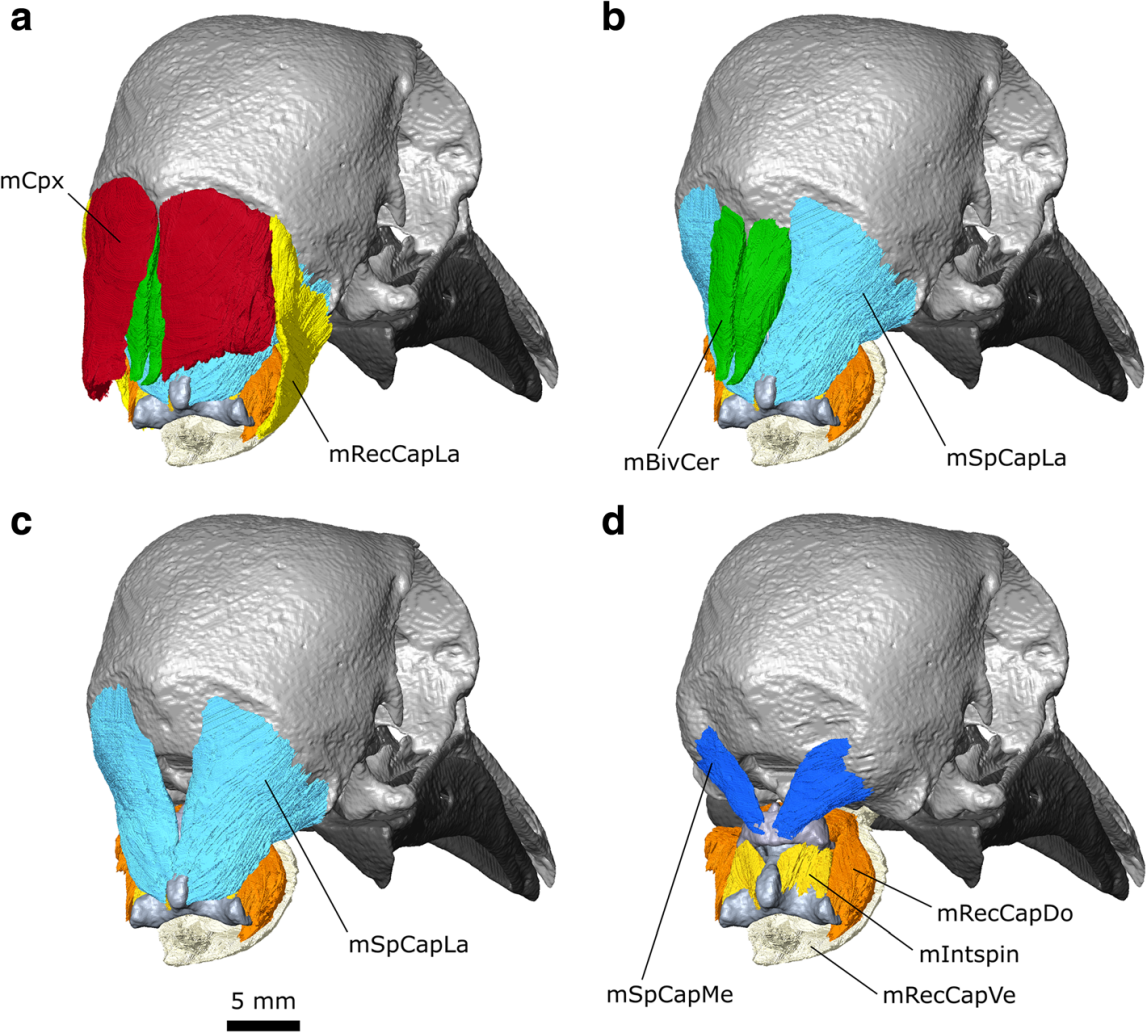
The anterior neck muscles of *Columba livia* in dorsal view. **a** to **c** represent increasingly deep dissections

**Path:** Converges into a wide sheet (Fig. 17a).

**Insertion:** A crescent-shaped insertion on the back of the skull superficial to all other neck muscles along the crista nuchalis (=occipital ridge) (Fig. 8b).

**Function:** Mediolateral and dorsal movements of the head.

#### m. Biventer Cervicis (m. BivCer)

The m. biventer cervicis of birds (e.g., [37, 108, 111]) is probably homologous with the m. Spinalis Capitis of lepidosaurians and the medial part of the M. Transversospinalis Capitis of crocodylians due to their similar points of origin and insertion [117].

**Origin:** From the neural spines of the posterior cervical vertebrae [37, 111, 117, 119]. The most posterior part present is narrow which might reflect its tendinous central portion [110].

**Path:** Passes anteriorly and forms two distinct bellies on either side of the midline posterior to the atlas and axis (Fig. 17a).

**Insertion:** On the posterior surface of the parietals, deep to the m. Cpx either side of the midline (Fig. 8b).

**Function:** Mediolateral and dorsal movements of the head.

#### m. Splenius Capitis Lateralis (m. SpCapLa)

The m. Splenius Capitis of birds may be composed of two parts [58, 111, 119]. The medial part is homologous to the m. Rectus Capitis Posterior of lepidosaurians and the m. Atloïdo-capitis of crocodylians [117], whereas the lateral part is homologous to the m. Obliquus Capitis Magnus of lepidosaurians and the m. Epistropheo-Capitis of crocodylians [117]. In *Columba livia* the two parts can be considered as separate muscles given that they have distinct sites of origin and insertion and are relatively easy to separate. An ascending branch of the CN XI passes between the ventral edges of the two parts before continuing into the body of the mSpCapL (Fig. 4b; Additional file 1).

**Origin:** From the anterodorsal surface of the axis (Fig. 17 b, c) rather than the third vertebra as found in *Podilymbus podiceps* [111].

**Path:** It passes anterolaterally and fans outwardly from the midline (Fig. 17c, d).

**Insertion:** It inserts ventromedial to the m. Cpx and lateral to the m. BivCer (Fig. 17c, d) and has a long sigmoid insertion (Fig. 8b).

**Function:** Mediolateral and dorsal movements of the head.

#### m. Splenius Capitis Medialis (m. SpCapMe)

The m. Splenius Capitis Medialis is smaller than the m. SpCapL and located deep to it rather than medial as in some other birds [58, 115].

**Origin:** It originates from the dorsal surface of the atlas (Fig. 17d) rather than the axis as found in *Podilymbus podiceps* [111].

**Path:** It passes anterolaterally and fans outwardly from the midline (Fig. 17c, d).

**Insertion:** It inserts ventromedial to the m. SpCapL and lateral to the m. BivCer and foramen magnum (Fig. 8b).

**Function:** Mediolateral and dorsal movements of the head.

#### m. Rectus Capitis Lateralis (m. ReCapLa)

The m. Rectus Capitis Lateralis of birds (e.g., [37, 108, 110]) is homologous to the dorsal part of the m. Rectus Capitis Anterior in lepidosaurians and the m. Iliocostalis Capitis of crocodylians [118]. It is broad and conspicuous in lateral view and appears to be innervated by CN XII.

**Origin:** The ventral processes of the axis (Fig. 14) as well as from the ventral surface of more posterior cervical vertebrae [37, 119].

**Path:** It passes dorsolaterally overlapping the m. Cpx close to its insertion but not as much as in *Buteo buteo* [58].

**Insertion:** The posterolateral edge of the cranium between the insertion for the m. SpCapLat and the m. DM (Fig. 14). It has a long slightly sigmoid insertion.

**Function:** Mediolateral movements of the head.

#### m. Rectus Capitis Ventralis (m. ReCapVe)

The m. rectus capitis ventralis is homologous to the ventral part of m. Rectus Capitis Anterior in lepidosaurians and the m. Rectus Capitis Anticus Major of crocodylians [118].

**Origin:** The medial part originates from the ventral surface of the atlas, axis, as well as more posteriorly located vertebrae [58]. The origin of the lateral part is not present in our specimen but in other taxa derives from the third, fourth, and possibly fifth vertebrae [58].

**Fig. 18:**
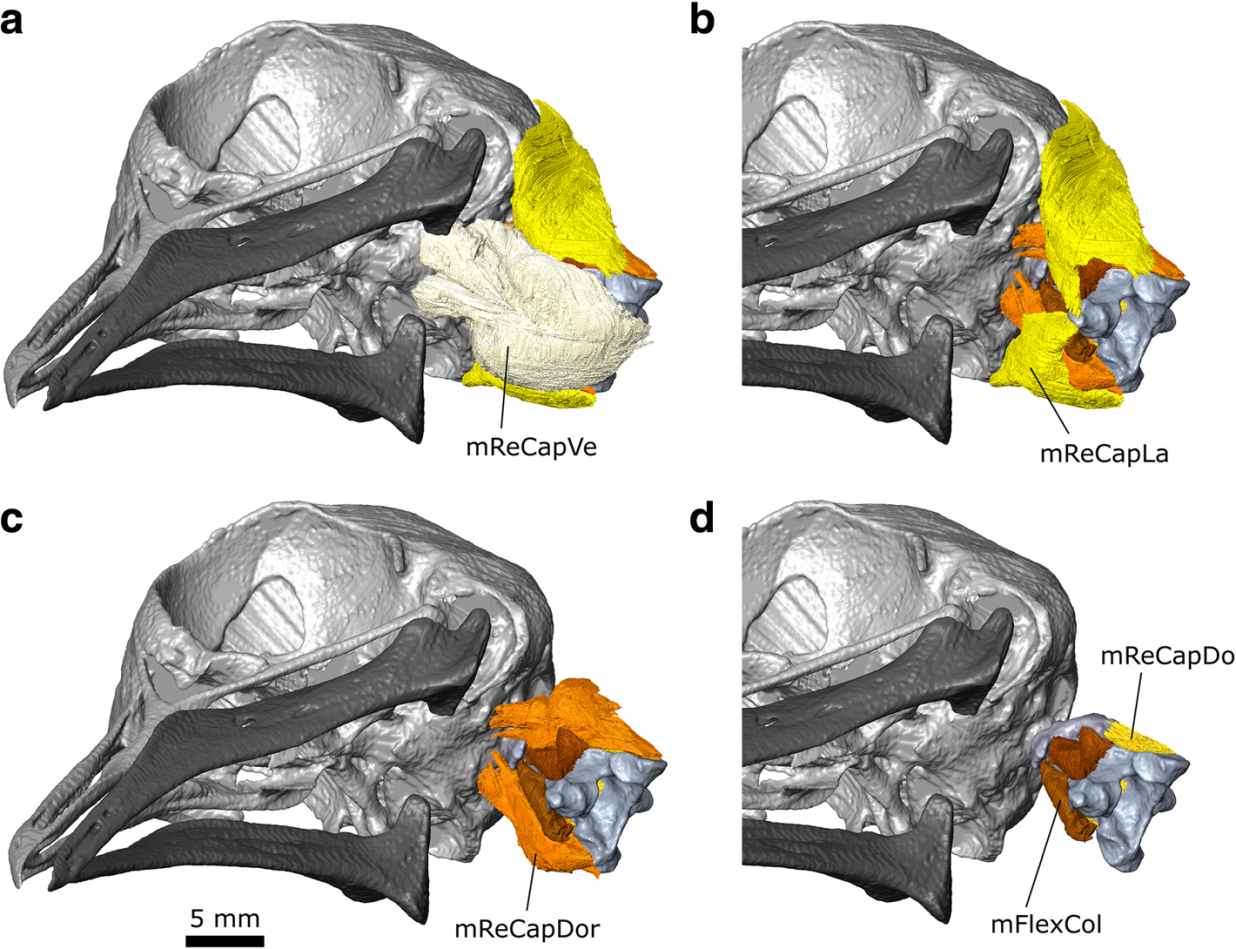
The anterior hypaxial muscles of *Columba livia* in ventral view. **a** to **c** represent increasingly deep dissections

**Path:** It passes rostrally ventral to the neck vertebrae forming two bellies (Fig. 18a).

**Insertion:** On the ventral surface of the basioccipital (Fig. 8b).

**Function:** Ventral movements of the head.

#### m. Rectus Capitis Dorsalis (m. ReCapDo)

The m. Rectus Capitis Dorsalis is one of the deepest hypaxial muscles [58].

**Origin:** The dorsal part originates from the posterolateral surface of the atlas, axis (Figs. 17d, 18b) as well as vertebrate located more posteriorly along the neck [119], often as far back as the 5th vertebra [110].

**Path:** It passes rostrally lateral to the atlas and axis.

**Insertion:** Three heads insert on the ventral surface of the cranium (Fig. 18b), posterior to the m. RecCapVe but medial to the exit of CN X and anterior to the exits of CN XII ([58]; Additional file 1). The three heads are also visible in *Eudromia elegans*, the elegant crested tinamou ([115]: Fig. 14).

**Function:** Ventral movement of the head.

#### m. Flexor Colli (m. FlexCol)

This deep epaxial muscle is relatively small [58].

**Origin:** This muscle originates from vertebrae that are located posterior to the axis [58, 111].

**Path:** It passes rostrally close to the midline ventral to the neck vertebrae (Fig. 18c).

**Insertion:** On the posteroventral surface of the atlas and possibly also the anterolateral surface of the axis (Fig. 18c).

**Function:** Contraction of this muscle would move the front of the neck posteroventrally.

#### m. Interspinales (m. Interspin)

There is clearly muscle spanning the dorsal halves of the atlas and axis, and this is likely part of the Interspinales [58]. There appears to be medial and lateral portions that are contiguous with one another (Additional file 1).

**Origin:** The medial portion originates from the lateral surface of the neural spine of the axis whereas the lateral part originates from the dorsal surface of the neural arch of the axis (Fig. 17d).

**Path:** The medial potion passes anterolaterally whereas the lateral portion passes anteroventrally.

**Insertion:** The medial portion inserts on the posterior surface of the posterolateral edge of the atlas whereas the lateral portion inserts on the posterolateral prominence of the atlas (Fig. 17d).

**Function:** Supporting connection between the axis and atlas.

## Conclusions

In this paper, we present a 3D digital dissection of the head of *Columba livia*, the rock dove, one of the most numerous, widespread and morphologically disparate avian species in the world, and use these data to describe its cranial and anterior cervical musculature. This was accomplished by applying the emerging technique of diceCT, combining contrast-enhancement of soft tissues using staining agents, CT scanning, and powerful visualization software. DiceCT has number of advantages over other methods, as it is non-destructive, replicable, and suitable for small and fragile specimens. It preserves the 3D topological relationships between anatomical structures and provides a powerful means of visualisation and communication that can provide an important tool to complement classical dissections (e.g., [30, 32, 33, 97, 120]). This approach is particularly good at providing the shape of the muscle and facilitating accurate communication regarding their relative positions to one another. It is also arguably easier for the anatomical work to be shared among a larger team of people [121]. However, there are also limitations such as the absence of colour, the difficulty interpreting aponeuroses, and occasionally problems inferring the precise origin/insertion of a muscle when it passes closely over a bony surface. Nevertheless, it is objectively more informative than a limited number of coronal and horizontal sections where the boundaries between individual muscles are not indicated (e.g., [122]).

Our interpretations and divisions of the jaw muscles most closely resemble those of van Gennip [33], in particular those relating to the structure of m. Adductor Mandibular Externus and m. Pterygoid Dorsalis. In contrast to previous studies (e.g., [30, 33]), we did not find a clear division of the m. Depressor Mandibulae and m. Pterygoideus Ventralis. However, we identify a division within the m. Adductor Mandibulae Externus pars Medialis not previously described. We were also able to successfully separate the m. Adductor Mandibulae Posterior from the m. Adductor Mandibulae Externus pars Medialis despite problems previously encountered [33]. As previous interpretations have suggested [31] the jaw muscles of *Columba livia* are less extensive that those of some other columbiform taxa [25, 30, 40] and this might reflect feeding behaviour related to dietary differences. Our digital dissection of the throat muscles corresponds to those of Bhattacharyya [30] and Zweers [32] and is consistent with the homologies inferred for chicken based on developmental observations [112]. Our interpretation of pigeon eye muscles clarifies the description of Chard and Gunlach [102] and highlights significant differences that exist between the eye muscles among avian taxa. We show that that muscle arrangement and relative size in *C*. *livia* are more similar to that of the turkey [108]), and particularly the tinamou [115] (Table 4), rather than that of the house sparrow [99]. How much these differences reflect phylogenetic inheritance, size, and lifestyle requires wider comparisons.

*Columba livia* is an important model organism across biological sciences and we will use these anatomical data for biomechanical analyses of the pigeon feeding system. This muscle arrangement can be simplified into a finite number of strands that enable explicit reporting of origins and insertions [47, 50]. It can also be used for multibody dynamics analysis to quantitatively test specific hypotheses regarding jaw movement and possibly gape [48, 51–53, 123] and coupled with Finite Element Analysis to examine the distribution of strain and structural deformation during feeding (e.g., [45, 59, 84, 124]). These approaches are highly amenable to sensitivity analyses and therefore extremely powerful for understanding the effects of parameters within models and the context of results. Moreover, the phylogenetic position of *C. livia* means that this study provides crucial data for reconstructing cranial tissues in extinct archosaurs [80, 81, 83], facilitating biomechanical analyses of morphological trends observed in the fossil record (e.g., [80]) or of novel skull structures lying outside the range of extant phenotypic diversity. (e.g., [59]).

Our digital dissection of *C*. *livia* (and those of *Buteo buteo* [58] and *Dacelo novaeguineae* [75]) provides a starting point for a wider digital survey of avian cranial muscle anatomy to compliment previous work e.g., [30, 33] and investigate patterns of evolution. The hyoid apparatus and eye muscles alone have great potential for valuable phylogenetic characters and as well as indicators of life habit that can be compared across clades (with independent contrasts). Now that the systematic relationships of birds are becoming increasing well understood [87, 88], and analytical tools are increasingly more powerful (e.g., [125–129]), there is great opportunity for a richer understanding of how phenotype might have underpinned their diversity and success.

Our 3D model is fully available, making it of use to other researchers, in anatomical, morphometric or taxonomic studies, as well as in education and outreach. Digital dissection is more palatable and accessible to the public than gross dissection, and provides an excellent starting point for students investigating comparative anatomy. The surface files from our digital dissection could be enlarged and printed in three dimensions to provide a further resource for research, communication, and teaching (e.g., [92, 130, 131]). The durable nature of such models means that they can be used repeatedly, and their colourful nature makes them attractive teaching or outreach tools, and a stepping stone to handling actual specimens. The kinaesthetic nature of the models has the potential to provide a more effective learning experience, particularly for those with sensory disabilities, especially given research suggesting that multi-sensory input (here = visual + tactile) improves memory performance [132].

## Additional files

Additional file 1: A digital dissection of the head of *Columba livia* as a three-dimensional pdf created using Tetra4D. (PDF 53101 kb)

Additional file 2: Terminology used for the hyoid skeleton. (XLSX 9 kb)

Additional file 3: Terminology used for the throat muscles. (XLSX 14 kb)

Additional file 4: A spreadsheet of the segmented structures, the extent of smoothing used, and final number of faces in the 3D pdf. (XLSX 42 kb)
