# Supplementary figures and images for: Digital dissection of the head of the rock dove (Columba livia) using contrast-enhanced computed tomography
Source: Zoological Lett. 2019 Jun 10;5:17. doi: 10.1186/s40851-019-0129-z (PMC6558907; doi:10.1186/s40851-019-0129-z)

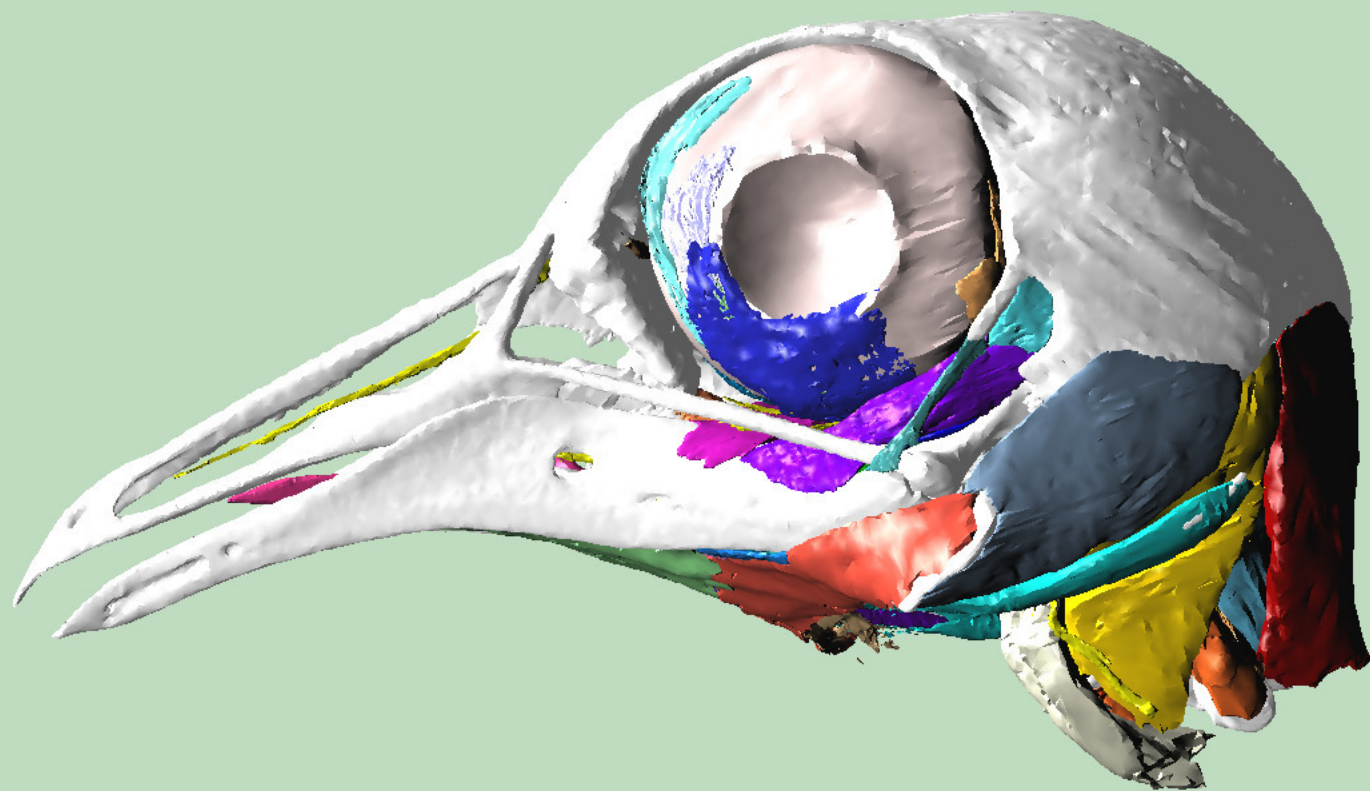

Supplement: Supplementary file 1 — A digital dissection of the head of Columba livia as a three-dimensional pdf created using Tetra4D. (PDF 53101 kb) [file 40851_2019_129_MOESM1_ESM.pdf]
